# Supplementary material for: Entropy-Enthalpy Compensations Fold Proteins in Precise Ways
Source: Int J Mol Sci. 2021 Sep 6;22(17):9653. doi: 10.3390/ijms22179653 (PMC8431812; doi:10.3390/ijms22179653)
Supplement: Supplementary file 1 [file ijms-22-09653-s001.zip › Supplementary1.pdf]

# Supplementary Information

**Table S1.** 1000 randomly selected small protein structures in PDB (PDBID).

| | | | | | | | | | |
|---|---|---|---|---|---|---|---|---|---|
| 134L | 1KP4 | 1Z3L | 3C3G | 4IC9 | 1FDD | 1R75 | 2OAI | 3OZZ | 5HBP |
| 155C | 1KSM | 1Z3M | 3C4S | 4IDL | 1FE3 | 1R9H | 2OJR | 3P2X | 5HBQ |
| 1A0B | 1KTH | 1Z3P | 3C5K | 4IP1 | 1FE5 | 1RAQ | 2OLI | 3PAZ | 5HDG |
| 1A18 | 1KXI | 1ZIA | 3C7I | 4IP6 | 1FER | 1RBI | 2ON8 | 3Q1D | 5HJC |
| 1A6F | 1KXW | 1ZIB | 3C97 | 4IPF | 1FES | 1RBW | 2OPY | 3Q4Y | 5HKN |
| 1AA2 | 1KXX | 1ZJ7 | 3CE8 | 4JHB | 1FEV | 1RBX | 2OQK | 3Q7Y | 5HMB |
| 1AB0 | 1KXY | 1ZPA | 3CEC | 4JJD | 1FF2 | 1RDS | 2OSN | 3QD7 | 5HPA |
| 1ACD | 1L5D | 208L | 3CQ1 | 4JZ3 | 1FIK | 1REX | 2OUB | 3R3K | 5HQI |
| 1ACF | 1L7L | 2A3G | 3CR2 | 4JZP | 1FKD | 1RFP | 2OUM | 3R5P | 5HVZ |
| 1AE2 | 1LAC | 2A7B | 3CR5 | 4K1V | 1FKF | 1RH2 | 2OXK | 3RH1 | 5I72 |
| 1AF5 | 1LCJ | 2A9I | 3CTG | 4K59 | 1FKK | 1RLK | 2OYZ | 3RHE | 5J6X |
| 1AHO | 1LEA | 2A9Q | 3CYI | 4KGT | 1FKV | 1RMD | 2OZ9 | 3RNT | 5JOE |
| 1ALB | 1LGP | 2AIF | 3DJN | 4KUO | 1FLQ | 1RNN | 2P0D | 3RSD | 5K2L |
| 1ANG | 1LKL | 2APB | 3DJU | 4KV4 | 1FLU | 1RNQ | 2P0F | 3RSK | 5KAZ |
| 1ANU | 1LOZ | 2AZ8 | 3DML | 4LBB | 1FLW | 1RNU | 2P1X | 3RTO | 5KM6 |
| 1AOJ | 1LPI | 2AZ9 | 3DP5 | 4LFQ | 1FLY | 1RNV | 2P2E | 3RVC | 5KUE |
| 1AQT | 1LRA | 2AZB | 3EAZ | 4LFS | 1FN5 | 1RNW | 2P61 | 3S3Y | 5KXF |
| 1AWJ | 1LSL | 2B1Y | 3ENU | 4LJN | 1FNJ | 1RNX | 2P64 | 3S8S | 5L8Z |
| 1AYC | 1LVE | 2B29 | 3ERS | 4LTT | 1FNK | 1RNZ | 2P6V | 3S9K | 5LAW |
| 1B0T | 1LXI | 2B4A | 3ETW | 4LYO | 1FOW | 1RRI | 2P8V | 3SGP | 5LAZ |
| 1B1E | 1LYO | 2B8G | 3EZM | 4MDQ | 1FOY | 1RRY | 2PAL | 3STM | 5LN2 |
| 1B1I | 1LZ4 | 2B9D | 3F3Q | 4MJJ | 1G2S | 1RS2 | 2PK7 | 3SUL | 5M9A |
| 1B1J | 1M1S | 2BEZ | 3F45 | 4ML2 | 1GBQ | 1RSI | 2PKT | 3T1X | 5MXY |
| 1B1U | 1M4A | 2BFH | 3F8C | 4MZ2 | 1GD6 | 1RTU | 2PNE | 3T3J | 5NGN |
| 1B2O | 1M4B | 2BHK | 3FAJ | 4N0Z | 1GDC | 1RWJ | 2PPI | 3T8R | 5NWX |
| 1B6E | 1M4M | 2BHO | 3FFY | 4N5T | 1GHJ | 1RZY | 2PPN | 3T8T | 5O3A |
| 1BAS | 1MB3 | 2BO1 | 3FRV | 4N6J | 1GKH | 1S3P | 2PVT | 3UAF | 5OC4 |
| 1BEA | 1MG6 | 2BPP | 3FWU | 4NEJ | 1GMG | 1S7I | 2PW5 | 3UB2 | 5OC8 |
| 1BEL | 1MH7 | 2BQQ | 3FYR | 4NXR | 1GOD | 1SDZ | 2PWS | 3UB3 | 5OMD |
| 1BFE | 1MH8 | 2BRF | 3FZ9 | 4OOH | 1GP3 | 1SF6 | 2PYK | 3UB4 | 5PAL |
| 1BGI | 1MK0 | 2BS5 | 3FZA | 4OV1 | 1GS3 | 1SF7 | 2PZW | 3UMD | 5PAZ |
| 1BHF | 1MKU | 2BTI | 3G7C | 4OXW | 1GSW | 1SFB | 2Q1M | 3UME | 5RHN |
| 1BKF | 1ML8 | 2BWK | 3GBQ | 4OZL | 1GV2 | 1SFG | 2QAS | 3UNN | 5RNT |
| 1BKV | 1MLI | 2BWL | 3GK2 | 4P15 | 1GVP | 1SKZ | 2QDB | 3V19 | 5TAB |
| 1BM2 | 1N9N | 2BZY | 3GK4 | 4P2P | 1GXT | 1SNP | 2QHE | 3V1G | 5U9U |
| 1BMG | 1N9O | 2CDS | 3GKY | 4P7U | 1H2P | 1SNQ | 2QHW | 3VXW | 5UEP |
| 1BO0 | 1NEH | 2COQ | 3GLW | 4P9E | 1HD0 | 1SSC | 2QIU | 3VYA | 5UER |
| 1BPQ | 1NEQ | 2CW4 | 3GM2 | 4P9V | 1HE7 | 1SV9 | 2QNW | 3WIT | 5UES |
| 1BQK | 1NKO | 2CXY | 3GM3 | 4PAZ | 1HEH | 1T00 | 2QR3 | 3WRP | 5UET |
| 1BQR | 1NLO | 2D4L | 3GQU | 4PE7 | 1HEY | 1T1D | 2QVT | 3WUN | 5UEV |
| 1BU3 | 1NN7 | 2D4M | 3GSP | 4PTA | 1HFX | 1T2J | 2R2Y | 3WVT | 5UEY |
| 1BXY | 1NNX | 2D58 | 3H33 | 4QYW | 1HIK | 1TAY | 2R34 | 3WW5 | 5USV |
| 1BYL | 1NXT | 2DB7 | 3H9W | 4RLM | 1HKF | 1TCY | 2R36 | 3WX4 | 5UVS |
| 1C0B | 1NXV | 2DH1 | 3HAF | 4RLN | 1HME | 1TDY | 2R39 | 3ZEK | 5UVY |
| 1C0C | 1O2E | 2DP9 | 3HAK | 4RUD | 1HQ8 | 1TEN | 2REA | 3ZK0 | 5UVZ |
| 1C76 | 1O3X | 2DRT | 3HEY | 4TQN | 1HQB | 1TGJ | 2REY | 4AHG | 5VEA |

| | | | | | | | | | |
|---|---|---|---|---|---|---|---|---|---|
| 1C9H | 1O41 | 2DU9 | 3HFF | 4TS8 | 1HRC | 1TNS | 2RKN | 4AHI | 5VR3 |
| 1C9V | 1O42 | 2DYQ | 3HFN | 4UNG | 1HRQ | 1TS9 | 2RLN | 4AHN | 5WOR |
| 1CDP | 1O43 | 2EFF | 3HJX | 4UNV | 1HSQ | 1TUW | 2RNS | 4AQI | 5WPB |
| 1CDT | 1O44 | 2EG2 | 3HNU | 4UTQ | 1I07 | 1TVQ | 2SAK | 4AQJ | 5WRB |
| 1CHN | 1O45 | 2EH9 | 3HQA | 4WBF | 1I0C | 1TXA | 2UUX | 4AYA | 5XUK |
| 1CI6 | 1O47 | 2EQL | 3HQB | 4WO6 | 1I0V | 1U36 | 2UZR | 4B9J | 5XWE |
| 1CKA | 1O4A | 2ES9 | 3I3Z | 4X1L | 1I3Z | 1U3J | 2V5F | 4BFN | 5YDN |
| 1CM3 | 1O4C | 2EYW | 3I7T | 4X1M | 1I9E | 1U3Y | 2V6H | 4BS7 | 5YI1 |
| 1COF | 1O4E | 2F3L | 3I7W | 4XC4 | 1IDI | 1U3Z | 2VJW | 4CGQ | 5YM7 |
| 1CS3 | 1O4G | 2F4G | 3I7X | 4XO2 | 1IGU | 1U42 | 2VKS | 4CPV | 5ZND |
| 1CUO | 1O4H | 2F4K | 3I7Y | 4XXL | 1IIZ | 1U68 | 2VP7 | 4CQI | 6B25 |
| 1CYI | 1O4I | 2FB0 | 3IE9 | 4Y92 | 1IJ0 | 1U9R | 2VSD | 4DH0 | 6BBK |
| 1D1M | 1O4J | 2FCD | 3IJU | 4YM8 | 1IJ1 | 1UHD | 2VSL | 4DH4 | 6BSY |
| 1DAQ | 1O4K | 2FD2 | 3IN2 | 4YOP | 1IJ2 | 1UIA | 2W1R | 4DP0 | 6CE6 |
| 1DCF | 1O4L | 2FKE | 3INC | 4YX5 | 1IKL | 1UIC | 2W51 | 4DP1 | 6CE8 |
| 1DDV | 1O4M | 2FKL | 3IRC | 4ZC3 | 1IOR | 1UID | 2WOR | 4DP2 | 6CEA |
| 1DDW | 1O4N | 2FLS | 3J4G | 5AEF | 1IOS | 1UIE | 2WOS | 4DP4 | 6CEC |
| 1DKJ | 1O4O | 2FLT | 3JTE | 5AFG | 1IOT | 1UIF | 2WQ0 | 4E1P | 6CED |
| 1DMM | 1O4Q | 2FMB | 3JZR | 5AI2 | 1IR7 | 1UIG | 2WQ3 | 4E1R | 6CEE |
| 1DMN | 1O4R | 2FO3 | 3K0X | 5B1F | 1IR8 | 1UKU | 2WQH | 4EC2 | 6CEF |
| 1DMQ | 1O5J | 2FQL | 3K63 | 5B1G | 1IR9 | 1UPJ | 2X44 | 4EES | 6DXH |
| 1DPY | 1O7Z | 2FTB | 3K6D | 5B3Q | 1IRQ | 1UPR | 2X9C | 4ET8 | 6DXR |
| 1DQ7 | 1OAP | 2GSP | 3K9I | 5B52 | 1IRW | 1UVY | 2XBD | 4ET9 | 6EKB |
| 1DYZ | 1OB6 | 2GV2 | 3KD0 | 5B79 | 1ISU | 1UW3 | 2XCZ | 4ETA | 6EVL |
| 1DZ0 | 1OD7 | 2H2B | 3KHQ | 5BMH | 1IYU | 1UW7 | 2XDY | 4ETB | 6EVM |
| 1E5B | 1ODA | 2H36 | 3KLU | 5BPO | 1IZA | 1UWM | 2XFE | 4ETD | 6FCE |
| 1E6K | 1OOI | 2H46 | 3KMJ | 5C39 | 1J2V | 1V07 | 2XKH | 4ETE | 6FGL |
| 1E6L | 1OPY | 2H8A | 3KQ6 | 5C4P | 1J4H | 1V46 | 2XMU | 4EWW | 6FGT |
| 1E6M | 1OR5 | 2H8V | 3KQI | 5C68 | 1J4I | 1VAT | 2YHN | 4EWZ | 6FGU |
| 1E97 | 1P0R | 2H95 | 3KTP | 5C6X | 1J73 | 1VED | 2YWZ | 4EX0 | 6FH6 |
| 1EA2 | 1P65 | 2HB4 | 3KU7 | 5CB9 | 1J7Z | 1VER | 2YXY | 4F1A | 6FH7 |
| 1ECW | 1P9G | 2HC8 | 3KVT | 5CO9 | 1J81 | 1VHF | 2Z44 | 4F2E | 6FXF |
| 1ED1 | 1PAL | 2HPL | 3L1M | 5CPV | 1J82 | 1VIH | 2Z7J | 4F68 | 6GF0 |
| 1EFQ | 1PAZ | 2HWV | 3L6W | 5CUL | 1JC7 | 1VMG | 2ZGD | 4F69 | 6H0K |
| 1EIF | 1PBI | 2I9V | 3LHC | 5D53 | 1JDL | 1W2L | 2ZHH | 4F6D | 6H0L |
| 1EIG | 1PI8 | 2IGP | 3LJM | 5D54 | 1JER | 1W6X | 3A0D | 4FC1 | 6HA4 |
| 1EMN | 1PKS | 2IIY | 3LLH | 5DKN | 1JHC | 1WAQ | 3A0S | 4FDX | 6I3S |
| 1EN2 | 1PO8 | 2INT | 3LMF | 5DM9 | 1JKD | 1WHI | 3A0V | 4FE6 | 6I5A |
| 1ENV | 1PZ4 | 2J5A | 3LR0 | 5DZD | 1JOI | 1WJG | 3A7L | 4G3O | 6IFH |
| 1EOQ | 1PZA | 2JIN | 3LVE | 5E0Z | 1JON | 1WJX | 3ADY | 4G4W | 6INS |
| 1EOT | 1PZB | 2JK7 | 3LYO | 5E4P | 1JPE | 1WRP | 3AZ5 | 4G4X | 6IQC |
| 1EOW | 1PZC | 2JP8 | 3LZ2 | 5E4X | 1JPO | 1WY9 | 3B7X | 4GBC | 6JQ4 |
| 1EQV | 1Q4R | 2JVH | 3M4T | 5EE2 | 1JZA | 1XW3 | 3B84 | 4GBN | 6MHN |
| 1EV3 | 1Q4V | 2KRI | 3MAZ | 5EH4 | 1K1Z | 1XW4 | 3BF2 | 4GFY | 6MPM |
| 1F1W | 1Q7O | 2LYO | 3MCD | 5EN9 | 1K40 | 1Y49 | 3BI9 | 4GQV | 6NF3 |
| 1FAO | 1Q8B | 2M6D | 3MF8 | 5ER4 | 1K58 | 1Y9T | 3BIA | 4GSP | 6PAZ |
| 1FAV | 1QFI | 2M6E | 3MYA | 5ET3 | 1K5A | 1YAT | 3BJO | 4HB6 | 6Q5K |
| 1FAZ | 1QKF | 2M6G | 3NGR | 5FD1 | 1KC2 | 1YEA | 3BKS | 4HE7 | 6QQJ |
| 1FB7 | 1QL2 | 2M6H | 3NRW | 5FG4 | 1KCQ | 1YEB | 3BOV | 4HMB | 6RLX |
| 1FB8 | 1QLS | 2MSS | 3NZ3 | 5G25 | 1KH0 | 1YGT | 3BY5 | 4HP9 | 6RNT |
| 1FD2 | 1QPV | 2NUH | 3O70 | 5GSP | 1KH8 | 1YMV | 3BYR | 4HRS | 7PAZ |

| | | | | | | | | | |
|---|---|---|---|---|---|---|---|---|---|
| 1FD8 | 1QSU | 2O0P | 3O8W | 5H0Y | 1KJT | 1YV7 | 3BZS | 4HV2 | 8PAZ |
| 1FDA | 1QTO | 2O0Q | 3ONH | 5HBL | 1KM8 | 1YVS | 3C12 | 4I3I | 9RAT |
| 1FDB | 1R26 | 2O1N | 3OSE | 5HBO | 1KM9 | 1Z21 | 3C1R | 4IAS | 9RNT |

**Table S2.** Amino acid sequences of β-turns of the 1000 protein samples.

PDBID:1AA2

AGYPNV NFT RDG RPDLI KKSN TKL VDHP

PDBID:1ACF

NL GAVT LDG SAGF AG DDR GS

TSK NEKI

PDBID:1AOJ

NSS MKD ASG NNI TPE NSSE MKD

DDRR NASG

PDBID:1AYC

RRW HPNI VDG KSNPG NG TGDY LYGG

HHGQ KNG

PDBID:1BU3

AFSGILA AADS DQDK SAGA DSDG

PDBID:1CKA

DEE KKG KPEEQ DSEG

PDBID:1FB7

LWQR GG DTGA IG CG PTPVN

PDBID:1FB8

SLGT GGLVK RN KDQMS YSQERV PF

PDBID:1FES

CSGCSGA

PDBID:1FNK

RDT TPDL LSGWQYV VTG-GLKK

PDBID:1FOY

TFIT PDL SMG

PDBID:1GBQ

PDBID:1NXV

PD LMLPEID AD KPF

PDBID:1O42

EEW KI NAENPRG SETTKGA NAK LDSG SR

ADGLCHR

PDBID:1O4C

EEW KI NAENPRG SETTKGA NAK LDSG SR

ADGLCHR

PDBID:1O4J

EEW KI NAENPRG SETTKG NAK LDSG SR

ADGLCHR

PDBID:1O4N

SIQAEEW KI NAENPRG SETTKGA NAK LDSG SR

ADGLCHR

PDBID:1O4O

SIQAEEW KI NAENPRG SETTKGA NAK LDSG SR

ADGLCHR

PDBID:1OD7

PK LSG PADV VSNG DYS-GKR SGD DKRFRGR

KD DS DVKG

PDBID:1ODA

PADV DYS-GKR SGDPKLV RGR MDHK KD DS

DVKG

PDBID:1PKS

LYD REE HLG SDG

PDBID:1PO8

| | | | | | | |
|---|---|---|---|---|---|---|
| ADD | KRGD | DQN | NG | KNY | | |
| PDBID:1GVP | | | | | | |
| KPSQA | SRQG | NEYP | DEGQ | GQFG | DRL | |
| PDBID:1J2V | | | | | | |
| EG | YDVP | NED | | | | |
| PDBID:1J4I | | | | | | |
| DGRT | KRG | EDG | GKQEVI | SVG | ATG | PGI |
| PPH | | | | | | |
| PDBID:1J82 | | | | | | |
| NLTKDR | CKNG | TGSS | YP | GNPY | | |
| PDBID:1K5A | | | | | | |
| DAKPQGR | LTSPCKD | ENKN | REN | WPPC | NG | QSA |
| PDBID:1LEA | | | | | | |
| FRS | VSGAS | | | | | |
| PDBID:1LGP | | | | | | |
| RLGAEE-GE | KR | RRGCD | DEKSG | STSG | VKKQ | QTG |
| YRKNE | | | | | | |
| PDBID:1MH8 | | | | | | |
| VPAR | GCY | QG | KGRN | NI | | |
| PDBID:1MK0 | | | | | | |
| TLNN | KD | HSS | GNV | PYE | NSKIN | |
| PDBID:1NLO | | | | | | |
| LYD | TET | KKG | NTEGD | LTTG | | |
| PDBID:1NNX | | | | | | |
| RDD | DD | AS | WNGV | TPKD | WN | |
| PDBID:1O4E | | | | | | |
| EEW | KI | NAENPRG | SETTKGA | NAK | LDSG | SR |
| ADGLCHR | | | | | | |
| PDBID:1OPY | | | | | | |
| DPFGQ | SHNG | NG | DEHG | | | |
| PDBID:1P9G | | | | | | |

| | | | | | | |
|---|---|---|---|---|---|---|
| GCY | IDGC | EP | DSKD | | | |
| PDBID:1PZA | | | | | | |
| AE | PA | NPG | VDK | IKDM | PEGA | KINE |
| CTPH | GDSP | | | | | |
| PDBID:1PZB | | | | | | |
| AE | PA | NPG | VDK | IKDM | PEGA | KINE |
| CTPH | GDSP | | | | | |
| PDBID:1RNQ | | | | | | |
| DSSTS | NLT-KDR | CKNG | TGSS | YP | GNPY | |
| PDBID:1RNV | | | | | | |
| NLTKDR | CKNG | TGSS | YP | GNPY | | |
| PDBID:1RNX | | | | | | |
| DSSTS | NLT-KDR | CKNG | TGSS | YP | GNPY | |
| PDBID:1RRI | | | | | | |
| YNR | ENPPI | | | | | |
| PDBID:1UIG | | | | | | |
| LDN | NF | TQA | NTDG | GILQ | SRWW | DGRTPGS |
| NLCN | SSD | DGN | KGTD | RGC | | |
| PDBID:1UW7 | | | | | | |
| PNN | TTQTAC | NSKGG | HQDL | KSDG | TPKG | KGL |
| PDBID:1WJG | | | | | | |
| FK | PDYL | GTRG | SLFL | | | |
| PDBID:2BEZ | | | | | | |
| NVLY | ISGI | NESL | | | | |
| PDBID:2BPP | | | | | | |
| IPSS | NN | GCY | KVL | NPYTN | NN | SSEN |
| NL | | | | | | |
| PDBID:2BTI | | | | | | |
| VG | GDE | GN | PKEV | VG | GDE | GN |
| PKEV | | | | | | |
| PDBID:2CXY | | | | | | |

| | | | | | | |
|---|---|---|---|---|---|---|
| CPRP | NAG | IYG | GAGN | | | |
| PDBID:1RDS | | | | | | |
| GS | DD | DYEG | MSDY | GDD | HTGASGDD | |
| PDBID:1S3P | | | | | | |
| AADS | GLKKK | DKDK | SSDA | DKDG | | |
| PDBID:1SKZ | | | | | | |
| PEGS | IITD | CPH | SRYG | LEP | PEG | RLTN |
| IDI | CPN | DKLG | | | | |
| PDBID:1T1D | | | | | | |
| SG | FPDT | PLRN | GGR | PVNV | | |
| PDBID:1TGJ | | | | | | |
| WKW | PK | PYLRSA | NPEAS | GR | | |
| PDBID:1TS9 | | | | | | |
| WIG | SPN | VG | TQN | TEKG | KRGR | KG |
| PDBID:1UPJ | | | | | | |
| LWQRP | GG | DTGA | GIGG | CG | TPVN | QIG |
| PDBID:1UVY | | | | | | |
| NGI | GPNA | ANM | | | | |
| PDBID:1VIH | | | | | | |
| HK | GKSG | SEKS | | | | |
| PDBID:2AIF | | | | | | |
| ADAEP | KDGS | | | | | |
| PDBID:2AZ9 | | | | | | |
| LWKRP | GG | DTGA | GIGG | CG | GPT | QIG |
| PDBID:2BHK | | | | | | |
| WDDW | PL | EFPL | DPEST | DSAN | | |
| PDBID:2BHO | | | | | | |
| NEND | DGH | HQQ | LGAD | FGEHWPA | DG | LVGL |
| PDBID:2BO1 | | | | | | |
| ARNT | AGES | | | | | |
| PDBID:2EFF | | | | | | |

| | | | | | | |
|---|---|---|---|---|---|---|
| GK | PEV | | | | | |
| PDBID:2DH1 | | | | | | |
| QGDV | LSNG | NLNKVNS | SSATG | IKDY | PEDT | PAGS |
| GGGTLGV | DTKG | NSEYN | VSGA | DSSTK | NDNG | SLGG |
| QGDV | LSNG | NLNKVNS | SSATG | IKDY | PEDT | PAGS |
| GGGTLGV | DTKG | NSEYN | VSGA | DSSTK | NDNG | SLGG |
| SEGNPAI | QGDV | LSNG | NLNKVNS | SSATG | PEDT | PAGS |
| GGGTLGV | DTKG | NSEYN | VSGA | DSSTK | NDNG | SLGG |
| SEGNPAI | QGDV | LSNG | NLNKVNS | SSAT | IKDY | PEDT |
| PAGS | GGGTLGV | DTKG | NSEYN | VSGA | DSSTK | NDNGD |
| DLKAKL | SLGG | | | | | |
| PDBID:2FD2 | | | | | | |
| IKCK | EVCPVD | PN | HPDEC | EDEV | DGVKGK | |
| PDBID:2FLT | | | | | | |
| QDRLT | PAGN | GG | VEYG | | | |
| PDBID:2LYO | | | | | | |
| LDN | NF | TQA | NTDG | GILQ | SRWW | DGRTPGS |
| NLCN | SSD | DGN | KGTD | IRGC | | |
| PDBID:2O1N | | | | | | |
| GCY | GWG | NG | LY | | | |
| PDBID:2OUM | | | | | | |
| KELA | LPHG | DKTG | ASF | AHK-PEGA | TF | TMG |
| PDBID:2P0D | | | | | | |
| GG | GN | LRGA | GRHLS | RRN | TIPG | SDH |
| PDBID:2PPN | | | | | | |
| DGRT | KRG | EDG | GKQEVI | SVG | ATG | PGI |
| PPH | | | | | | |
| PDBID:2QR3 | | | | | | |
| NHFS | MNFTSNE | YRDL | PW | | | |
| PDBID:2UZR | | | | | | |
| GKYIK | NDG | KER | VAQ | ERPRPN | WT | |

| | | | | | | |
|---|---|---|---|---|---|---|
| GG | ENG | KQG | GD | DRSG | | |
| PDBID:2FCD | | | | | | |
| DKKG | KD | DSSLRDAS | | | | |
| PDBID:2H46 | | | | | | |
| HPW | KI | HDG | ESAPG | GN | DGAG | RST |
| SRNQ | | | | | | |
| PDBID:2HC8 | | | | | | |
| DG | AVG | RPG | KG | FGA | | |
| PDBID:2HPL | | | | | | |
| NPSD | IGN | LPVRG | GET | PKKA | | |
| PDBID:2JVH | | | | | | |
| AMVSI | KDS | PMDV | | | | |
| PDBID:2M6H | | | | | | |
| GCPDP | | | | | | |
| PDBID:2QNW | | | | | | |
| NPES | | | | | | |
| PDBID:2VSD | | | | | | |
| PSQG | SLG | PRM | NG | DKEQ | SEPL | |
| PDBID:2YWZ | | | | | | |
| PR | ETGE | LGS | GR | KGSK | SDL | SPSCYSYP |
| PDBID:2ZGD | | | | | | |
| DVAA | DKNG | DKFG | | | | |
| PDBID:3BJO | | | | | | |
| LDYI | EE | FKGKYE | PQRG | QS | | |
| PDBID:3C1R | | | | | | |
| NE | KTYC | KLK | TVP | NG | PIL | |
| PDBID:3CE8 | | | | | | |
| ND | LEFL | RE | KHN | | | |
| PDBID:3DJU | | | | | | |
| NHK | PSE | PY | GEDG | | | |
| PDBID:3F3Q | | | | | | |

| | | | | | | |
|---|---|---|---|---|---|---|
| PDBID:2V5F | | | | | | |
| DPEH | HHHH | | | | | |
| PDBID:2W1R | | | | | | |
| RD | GS | RS | SDA | NG | NG | KDQ |
| PDBID:3F45 | | | | | | |
| TAAD | DKDK | SSDA | DKDG | | | |
| PDBID:3FZ9 | | | | | | |
| NP | KTWC | TVP | GG | | | |
| PDBID:3HFF | | | | | | |
| DGP | KESNG | HEEV | DKDG | SVI | GDH | IIG |
| DLGK | | | | | | |
| PDBID:3HFN | | | | | | |
| VTG | DPT | DRQ | KQA | SLP | VTGD | DPT |
| DRQ | | | | | | |
| PDBID:3KD0 | | | | | | |
| AGDK | ATWC | SN | VDDS | SMP | KG | |
| PDBID:3LYO | | | | | | |
| LDN | NF | TQA | NTDG | GILQ | SRWW | DGRTPGS |
| NLCN | SSD | DGNGMNAW | KGTD | RGC | | |
| PDBID:3O8W | | | | | | |
| SDD | KFGD | IRTG | | | | |
| PDBID:3Q4Y | | | | | | |
| VPSR | GCY | KG | QG | KGDN | NI | |
| PDBID:3RVC | | | | | | |
| MLI | GP | QAI | FD | EEG | LPSL | |
| PDBID:3UB4 | | | | | | |
| SRWSKD | GAI | QALS | PGAEG | LGL | GRG | |
| PDBID:4AHN | | | | | | |
| DNS | DAKPQGR | TSPCKD | ENKN | RENL | WPPC | NG |
| QSIFH | | | | | | |
| PDBID:4BFN | | | | | | |

| | | | | | | |
|---|---|---|---|---|---|---|
| HHH | ATWC | YPQA | VDEL | AMP | NG | |
| PDBID:3GM2 | | | | | | |
| RTDD | VM | AVT | | | | |
| PDBID:3H33 | | | | | | |
| TRIG | IDGF | GKGC | | | | |
| PDBID:3HAF | | | | | | |
| VGGLG-GY | | | | | | |
| PDBID:3K6D | | | | | | |
| NQ | VSDRIPGS | NENSG | DREAIP | DENG | | |
| PDBID:3KQI | | | | | | |
| GSMA | CVCR | DVTR | CDACK | KKKR | | |
| PDBID:3KTP | | | | | | |
| HPT | NWPP | PG | WKGL | | | |
| PDBID:3MCD | | | | | | |
| ERTL | LPYM | ERTL | LPY | | | |
| PDBID:3NRW | | | | | | |
| TDA | ARE | DEDL | | | | |
| PDBID:3T8R | | | | | | |
| KEGQ | RGAKG | RVP | IESE | | | |
| PDBID:4B9J | | | | | | |
| KEG | PENV | VY | TADDSQ | DDKG | KD | TPN |
| PDBID:4CPV | | | | | | |
| FAGVLN | AADS | GLTSK | DQDK | FKADA | DSDG | |
| PDBID:4E1P | | | | | | |
| DDFDGSG | DG | DDFDG | DG | | | |
| PDBID:4F2E | | | | | | |
| NG | MGG | PE | KKSV | PDFG | PMG | GMNMM |
| PDBID:4G3O | | | | | | |
| FPQV | | | | | | |
| PDBID:4G4W | | | | | | |
| DYDS | NANS | KEAPV | | | | |

| | | | | | | |
|---|---|---|---|---|---|---|
| PSSAS | DGS | NIAY | DGSD | SGS | VKGI | SG |
| PDBID:4CQI | | | | | | |
| KEGSTV | | | | | | |
| PDBID:4DH0 | | | | | | |
| DGRT | KRG | EDG | GK | SVG | ATG | PGI |
| PPH | | | | | | |
| PDBID:4F69 | | | | | | |
| FGFKGV | | | | | | |
| PDBID:4G4X | | | | | | |
| DYDS | NANS | KEAPVNP | | | | |
| PDBID:4K1V | | | | | | |
| DPFGQ | TGP | SHNG | NG | DEHG | | |
| PDBID:4LBB | | | | | | |
| IPA | IAGE | QG | IPA | IAGE | QG | |
| PDBID:4MJJ | | | | | | |
| GL | TLNP | GI | EDE | | | |
| PDBID:4UNV | | | | | | |
| SLGQ | TSSDVGGY | HAGK | EVN | PSGVPDR | GN | SGL |
| SD | | | | | | |
| PDBID:4X1M | | | | | | |
| YKDSP | VPGK | DRSS | GG | LLQEF | SAPT | TDK |
| GV | | | | | | |
| PDBID:4Y92 | | | | | | |
| TTTD | KKG | NTEG | SLTTG | | | |
| PDBID:5B1F | | | | | | |
| LDN | NF | TQA | NTDG | GILQ | SRW | DGRTPGS |
| LCN | SSD | DGNGMNAW | KGT | RGC | | |
| PDBID:5B3Q | | | | | | |
| DNGLGN | GFN | YLTY | RPER | WKD | PEEDP | VTDLW |
| GH | | | | | | |
| PDBID:5EE | | | | | | |

| | | | | | | |
|---|---|---|---|---|---|---|
| | | NP | | | | |
| PDBID:4GBC | | | | | | |
| TSI | TSI | | | | | |
| PDBID:4I3I | | | | | | |
| AFGS | GDG | VIG | SPEF | DYQM | SGD | |
| PDBID:4NXR | | | | | | |
| TAAD | DG | KETG | KAG | NN | | |
| PDBID:4OOH | | | | | | |
| DSST | NLT-KDR | CKNG | TGSS | YP | GNPY | |
| PDBID:4X1L | | | | | | |
| ADG | YKDSP | VPGK | GG | LLQDGEF | KPT | TDK |
| GV | | | | | | |
| PDBID:4ZC3 | | | | | | |
| DMN | | | | | | |
| PDBID:5AI2 | | | | | | |
| KICG | EDA | SPGT | PDDW | CPICG | | |
| PDBID:5C68 | | | | | | |
| PD | PG | AEPP | GAGE | GPGG | | |
| PDBID:5D54 | | | | | | |
| TSI | FVNQ | | | | | |
| PDBID:5DZD | | | | | | |
| PEG | TVDG | HNRR | PRTG | PEG | TVDG | HNRR |
| PRTG | | | | | | |
| PDBID:5E4X | | | | | | |
| GDDGKT | MSDA | PQDNV | | | | |
| PDBID:5H0Y | | | | | | |
| SP | AVRG | AIN | AADD | SESG | TEEEFV | PFYE |
| NDSG | PY | | | | | |
| PDBID:5I72 | | | | | | |
| PQF | KSCW | NKG | CNNH | CPICK | PQF | KSCWF |
| NKG | CNNH | CPICK | | | | |
| PDBID:5JO | | | | | | |

| | | | | | | |
|---|---|---|---|---|---|---|
| 2 | | | | | | |
| NG | FNSN | SDY | GP | GG | EV | DGD |
| LDD | | | | | | |
| PDBID:5HBL | | | | | | |
| IPE | HE | QAG | APA | ED | | |
| PDBID:5HBQ | | | | | | |
| IPE | HE | DQAG | APA | ED | | |
| PDBID:5HJC | | | | | | |
| YKP | IIKHP | PPDH | | | | |
| PDBID:5HPA | | | | | | |
| IPE | HE | QAG | APA | ED | | |
| PDBID:5HVZ | | | | | | |
| PER | PER | | | | | |
| PDBID:5KM6 | | | | | | |
| KEIP | DDR | DISP | AP | LNKG | QSVY | HWPP |
| PDBID:5KUE | | | | | | |
| NG | PS | KPISD | DRGI | SPDG | ND | |
| PDBID:5KXF | | | | | | |
| GG | PEVV | LN | LDDG | QER | | |
| PDBID:5M9A | | | | | | |
| DAKPQGR | GLTSPCKD | ENKN | REN | GGSPWPPC | NG | |
| PDBID:5YDN | | | | | | |
| TADG | ESQP | | | | | |
| PDBID:6CE8 | | | | | | |
| CPH | PAAG | DVTQ | CGDCG | IQE | LSCY | GRYING |
| YIDL | YYCQ | | | | | |
| PDBID:6CEA | | | | | | |
| PWCPH | PAAG | DVTQ | CGDCG | IQE | LSCY | GRYING |
| YIDL | YYCQ | | | | | |
| PDBID:6FGU | | | | | | |
| SMSV | HED-AWPFL | NLKLVPG | IKKP | EDDS | | |
| PDBID:6GF | | | | | | |

| | | | | | | |
|---|---|---|---|---|---|---|
| E | | | | | | |
| LKD | YPEI | GT | SDK | GD | KN | GP |
| PDBID:5L8Z | | | | | | |
| TDVL | AGF | NPSTG | | | | |
| PDBID:5RHN | | | | | | |
| RPGG | KEIP | DDQ | DISP | AP | KKH | LKKG |
| QSVY | NWPP | | | | | |
| PDBID:5RNT | | | | | | |
| CD | GS | GSNS | NYEGFDF | LSSG | ENN | HTGASGNN |
| PDBID:5UEY | | | | | | |
| AKK | DVEALG | IKHP | PPDH | | | |
| PDBID:5USV | | | | | | |
| TSI | FVNQ | | | | | |
| PDBID:5YM7 | | | | | | |
| EDKSPDS | GK | | | | | |
| PDBID:6B25 | | | | | | |
| PQENE | RPG | NED | QD | ANF | QQNE | VRT |
| KEN | DG | GK | | | | |
| PDBID:6IQC | | | | | | |
| KIP | | | | | | |
| PDBID:9RAT | | | | | | |
| DSST | NLT-KDR | CKNG | YP | GNPY | | |
| PDBID:9RNT | | | | | | |
| CD | GS | GSNS | NYEGFDF | LSSG | ENN | HTGASGNN |
| PDBID:1AE2 | | | | | | |
| KPSQA | SRQG | NEYP | DEGQ | GQFG | DRL | |
| PDBID:1AHO | | | | | | |
| DDVN | PY | PDHV | | | | |
| PDBID:1B1U | | | | | | |
| TPSG | RLLQ | PG | LVTEVECN | | | |
| PDBID:1BHF | | | | | | |

| | | | | | | |
|---|---|---|---|---|---|---|
| 0 | | | | | | |
| LDN | NF | TQA | NTDG | GILQ | SRW | DGRTPGS |
| LCN | SSD | NGMNAW | KGTD | RGC | | |
| PDBID:6HA4 | | | | | | |
| KSKN | NDAG | KDN | TYNN | | | |
| PDBID:7PAZ | | | | | | |
| AE | PA | NPG | VDK | IKDM | PEGA | KINE |
| CTIH | GDSP | | | | | |
| PDBID:1ANU | | | | | | |
| SVG | GVPSKG | DPNV | GDII | DPN | NDL | |
| PDBID:1AWJ | | | | | | |
| EETL | DPQE | DKNGHE | SSYL | | | |
| PDBID:1B1E | | | | | | |
| QDN | DAKPQGR | TSPCQD | ENKN | REN | GGS | WPPC |
| NG | | | | | | |
| PDBID:1BEA | | | | | | |
| GPDA | PG | | | | | |
| PDBID:1BMG | | | | | | |
| RHP | DG | PP | NG | KS | KDW | NSKD |
| VTLEQP | | | | | | |
| PDBID:1CHN | | | | | | |
| WNMP-NMD | DGAMSAL | EA | KEN | PF | | |
| PDBID:1COF | | | | | | |
| KKYK | NDAK | PEND | NGNE | PDTA | LNG | DFSEVS |
| RG | GA | | | | | |
| PDBID:1DMN | | | | | | |
| DPFGQ | SHNG | NG | DEHG | | | |
| PDBID:1E6K | | | | | | |
| MPNMD | DGAMSAL | EAK | PF | | | |
| PDBID:1FD2 | | | | | | |
| IKCK | EVAPVD | PN | HPDEC | CPAQ | DGVKG | |

| | | | | | | |
|---|---|---|---|---|---|---|
| EPW | KNL | APGNTHG | SES-TAGS | DQNQG | LDNG | PR |
| SDGLCTR | | | | | | |
| PDBID:1BQR | | | | | | |
| KD | PA | APG | TDK | IKGM | PDGA | KINE |
| CTPH | GDAP | | | | | |
| PDBID:1CM3 | | | | | | |
| TPNGLD | NG | AKS | TQG | GED | | |
| PDBID:1ECW | | | | | | |
| SVLS | LRPGG | TGTA | | | | |
| PDBID:1EIG | | | | | | |
| VSKR | PENR | RSTC | KKG | DPKQ | KK | |
| PDBID:1FD8 | | | | | | |
| VM | CSG | PD | LEKQ | | | |
| PDBID:1FDB | | | | | | |
| IKCK | CPVD | PN | HPDEC | CPAQ | EVW | DGVKGK |
| PDBID:1FKK | | | | | | |
| PGDGRT | KRG | EDG | SRDRN | GKQEVI | SVG | ATG |
| PGI | PPN | | | | | |
| PDBID:1FNJ | | | | | | |
| RDT | TPDL | LSGWQYV | VTG-GLKK | PQDQI | | |
| PDBID:1GDC | | | | | | |
| CLVCS | YG | CAGRN | NLEA | | | |
| PDBID:1IIZ | | | | | | |
| AR | TDK | NKNG | GLFQ | DKYW | GST | GKDCN |
| TDD | KFDAW | NHS | | | | |
| PDBID:1IKL | | | | | | |
| CQCIKT | HPKF | GPHCA | SDG | DPKE | | |
| PDBID:1IRQ | | | | | | |
| PDK | | | | | | |
| PDBID:1IYU | | | | | | |

| | | | | | | |
|---|---|---|---|---|---|---|
| PDBID:1FN5 | | | | | | |
| LDN | NF | TQA | NTDA | GILQ | SRWW | DGRTPGS |
| NLCN | SSD | DGNGMNAW | KGTD | RGC | | |
| PDBID:1HD0 | | | | | | |
| SWDT | PITG | NG | | | | |
| PDBID:1HIK | | | | | | |
| HKCD | TLCTEL | DIFAASKN | DTRCL | PVK | | |
| PDBID:1HRQ | | | | | | |
| PAN | AADD | QDATKS | QF | ADAAGA | DE | QL |
| VNG | CASW | | | | | |
| PDBID:1IR9 | | | | | | |
| LDN | NF | TQA | NTDG | GILQ | SRWW | DGRTPGS |
| NLCN | SSD | DGN | KGTD | RGC | | |
| PDBID:1JER | | | | | | |
| VG | PSSP | VG | PANA | FVNS-DND | VGTH | |
| PDBID:1KCQ | | | | | | |
| RRV | NNGD | GN | GSNS | RSGR | EGTE | |
| PDBID:1L7L | | | | | | |
| ANNEAG | NPGD | YGP | REH | NS | VNT | PNNV |
| VPGTYGNN | | | | | | |
| PDBID:1LZ4 | | | | | | |
| MDG | GY | TRA | AGDR | GIFQ | SRY | DGKTPGA |
| AAH | QDN | DPQ | QNR | VQGC | | |
| PDBID:1M4B | | | | | | |
| NYKNPK | KKA | | | | | |
| PDBID:1M4M | | | | | | |
| QIW | FKNW | CACT | TENEPDL | FFCF | EPDD | SPGC |
| VKK | | | | | | |
| PDBID:1ML8 | | | | | | |
| MQA | GL | ASG | GNSGDKA | QD | RDL | |

KTG EVEQ SAKA SPKA KLGD EG PAAGA

PDBID:1J81

NLTKDR CKNG TGSS YP GNPY

PDBID:1K40

PAS

PDBID:1LKL

EPW KNL APGNTHG SES-TAGS QNQ LDNG PR

SDGLCTR

PDBID:1LRA

CD GS GSNS NYEG LSSG ENN HTGASGNN

PDBID:1N9O

ATLPDC GPDEVLG GEGT RKDG TPDG

PDBID:1NEQ

NEKARD LER IWPSRYQ

PDBID:1NKO

EGM DSD NPAW RDR RDA GN

PDBID:1O4M

EEW KI NAENPRG SETTKGA NAK LDSG SR

ADGLCHR

PDBID:1O7Z

CIS NPRS SQFCP KKKGE NPES CISI SQFCP

KKGE NPES

PDBID:1P65

SDSG SDSG

PDBID:1PZ4

GIRM PA GG LKNV AE GALP

PDBID:1QLS

RDGNNT TE DLDS

PDBID:1R9H

TPKK GG TTG ENG GRGNVI DA PPK

GG

PDBID:1RBX

KAV

PDBID:1N9N

ATLPDC GEGT RKDG TPDG

PDBID:1OOI

FNF NKKG ADGQ

PDBID:1P0R

RLG NTDD QTG RWNKI KWYT KDHV LGDYE

HDG

PDBID:1PAZ

AE PA NPG VDK IKDM PEGA KINE

CTPH GDSP

PDBID:1PBI

CDT SNPP SNPP QCHN KNKSA CDT SNPP

SNPP QCHN KN

PDBID:1QPV

KKYK NDAK NGNE PDTA LNGV DFSEVS

PDBID:1RNZ

DSSTS NLT-KDR CKNG TGSS YP GNPY

PDBID:1RRY

NVIDT YNR ENP

PDBID:1RSI

NVIDT YNR ENPPI

PDBID:1SFB

LDN RG NF TQA NTDG YGILQ NSRWWC

DGRTPGS NLCN LLSSDITA VSDG CKGT WIR

PDBID:1U3Z

DRT VTG KD EENG RQF YKDVNI RKSDL

PDBID:1U42

DRT CVTG QKDDI EGV SPTDV YKDVNI KSDL

PDBID:1UHD

REG VNNG KRGE AIG DEKN LNY

PDBID:1UKU

| | | | | | | |
|---|---|---|---|---|---|---|
| DSST | NLT-KDR | CKNGQTN | TGSS | YP | GNPY | |
| PDBID:1SF6 | | | | | | |
| GRCE-LAAA | LDN | RG | NF | TQA | NTDG | GILQ |
| SRWWC | DGRTPGS | NLCN | KGT | WIRGC | | |
| PDBID:1SF7 | | | | | | |
| LA | LDN | RG | NF | TQA | NTDG | GILQ |
| SRWW | DGRTPGS | NLCN | SSDI-TAS | | | |
| PDBID:1TNS | | | | | | |
| LPGLP | RAGVKG | GEI | TSLGYF | | | |
| PDBID:1U36 | | | | | | |
| DRT | VTG | KD | RQF | YKDVNI | RKSDL | |
| PDBID:1VER | | | | | | |
| PR | ETGE | DA | LGS | GR | KGSK | RDL |
| RLS | | | | | | |
| PDBID:1W6X | | | | | | |
| SKL | KAG | KD | RG | SKL | KAG | KD |
| RG | | | | | | |
| PDBID:1WRP | | | | | | |
| SPYS | | | | | | |
| PDBID:1XW3 | | | | | | |
| GAQG | PDL | | | | | |
| PDBID:1XW4 | | | | | | |
| GAQG | PDL | | | | | |
| PDBID:1YAT | | | | | | |
| PGDGAT | KTG | ENG | GV | SVG | PRG | PGL |
| PPN | | | | | | |
| PDBID:1ZIB | | | | | | |
| KD | PA | APG | TDK | IKGM | PDGA | KINE |
| CTPH | GDAP | | | | | |
| PDBID:1ZPA | | | | | | |
| LWKRP | GG | DTGA | IG | CG | GPTPVN | |

| | | | | | | |
|---|---|---|---|---|---|---|
| RLIA | EG | YDVP | | | | |
| PDBID:1UPR | | | | | | |
| DPNL | DSSGLR | GH | DSRE | LPSY | APRGRRF | PG |
| PDBID:1V07 | | | | | | |
| VN | FGFKGV | | | | | |
| PDBID:1V46 | | | | | | |
| NAFT | | | | | | |
| PDBID:1YV7 | | | | | | |
| TRDVD | HCK | KGI | TSRPCK | NQ | | |
| PDBID:2B4A | | | | | | |
| CD | VD | QTKQ | SSEH | KPF | | |
| PDBID:2B9D | | | | | | |
| CAYCE | CAYCE | | | | | |
| PDBID:2CDS | | | | | | |
| LDN | NF | TQA | NTDG | GILQ | SRW | DGRTPGS |
| LCN | SSD | DGN | KGTD | IRGC | | |
| PDBID:2DP9 | | | | | | |
| GG | KDE | RRPGR | LSEV | | | |
| PDBID:2EG2 | | | | | | |
| FDF | RVGD | IRTG | | | | |
| PDBID:2EQL | | | | | | |
| EMDG | NF | TRA | NANG | GLFQ | NKWW | KRSS |
| NACN | DPK | KDK | | | | |
| PDBID:2ES9 | | | | | | |
| IKN | STY | | | | | |
| PDBID:2GSP | | | | | | |
| CD | GS | GSNS | NYEG | LSSG | ENN | HTGASGNN |
| PDBID:2H8V | | | | | | |
| VNE | SL | DDEA | LPT | | | |
| PDBID:2INT | | | | | | |
| TLCTEL | DIFAASKN | DTRCL | | | | |

| | | | | | | |
|---|---|---|---|---|---|---|
| PDBID:2A3G | | | | | | |
| ASV | | | | | | |
| PDBID:2BS5 | | | | | | |
| GTVP | NG | DGK | GD | GS | GT | DGNG |
| PDBID:2D58 | | | | | | |
| SSD | NGNG | | | | | |
| PDBID:2DB7 | | | | | | |
| SGGYFD | DASD | DASD | | | | |
| PDBID:2DU9 | | | | | | |
| GTLS | RGI | | | | | |
| PDBID:2FLS | | | | | | |
| NC | KTSC | TVP | NG | | | |
| PDBID:2OSN | | | | | | |
| YIN | GCYC | QP | RTAD | MISASNSC | | |
| PDBID:2P8V | | | | | | |
| AR | DPATK | ATRN | GA | TPNM | SQK | |
| PDBID:2PAL | | | | | | |
| SFAG | AADS | DQDK | SPSA | DKDG | | |
| PDBID:2QIU | | | | | | |
| FVN | TSI | NY | GERG | | | |
| PDBID:2R39 | | | | | | |
| DP | DRNQ | FRVAGE | LNDV | EPG | SA | |
| PDBID:2WQH | | | | | | |
| YPNN | YPNN | | | | | |
| PDBID:2XBD | | | | | | |
| WSD | SSAW | NGSQ | NA | SGST | PNGS | KNGS |
| PDBID:3C97 | | | | | | |
| TN | FD | DIQMPVMD | IDDD | GAEL | KPL | |
| PDBID:3CR2 | | | | | | |
| EGDKH | SHFL | DSDG | | | | |
| PDBID:3FWU | | | | | | |
| PEDLV | DST | FG | LGG | PLHC | NG | |

| | | | | | | |
|---|---|---|---|---|---|---|
| PDBID:2KRI | | | | | | |
| VKKA | QG | KNG | LHG | KEKK | DG | PKCF |
| SSLAF | DASDV | GPA | CNSS | ACD | CEDG | |
| PDBID:2OYZ | | | | | | |
| HG | QAP | VGE | SG | EGN | | |
| PDBID:2P2E | | | | | | |
| DSN | SLN | DYKKDYN | NI | NTNN | DNS | |
| PDBID:2RLN | | | | | | |
| NLTKDR | CKNG | TGSS | YP | GNPY | | |
| PDBID:2XCZ | | | | | | |
| SG | IGA | NG | | | | |
| PDBID:3B7X | | | | | | |
| QRM | ISGDRG | APDA | EHM | LED | TLG | PPL |
| PPN | | | | | | |
| PDBID:3CR5 | | | | | | |
| EGDKH | SHFL | DSDG | | | | |
| PDBID:3ETW | | | | | | |
| NTRFY | | | | | | |
| PDBID:3F8C | | | | | | |
| NGEME | DEGR | | | | | |
| PDBID:3FRV | | | | | | |
| PPK | GQ | | | | | |
| PDBID:3H9W | | | | | | |
| WQT | HRDG | DDSG | | | | |
| PDBID:3HQA | | | | | | |
| KH | NNDE | NNDE | | | | |
| PDBID:3IRC | | | | | | |
| TQHG | DAP | EKG | TANP | KE | GEKA | |
| PDBID:3L6W | | | | | | |
| PEKGDEK | DWTQ | IRSIN | GTDSD | GAKE | TDDH | KYDHT |
| DASVL | RPNNAVF | LPPK | KRGT | SVDEAVT | PPFS | LGKM |
| PEKGDEK | DWTQ | IRSIN | KSDD | GAKE | TDDH | KYDHT |

| | | | | | | |
|---|---|---|---|---|---|---|
| PDBID:3K0X | | | | | | |
| KDG | NG | GS | VTVVLPDV | QKH | DG | AVGI |
| PDBID:3K63 | | | | | | |
| RLKDT | AA | PNQ | YQNE | YPDG | KKNN | NPYG |
| DENG | PG | | | | | |
| PDBID:3K9I | | | | | | |
| AS | FPNH | DKLDE | | | | |
| PDBID:3KHQ | | | | | | |
| CSQ | PR | KRSS | RGSQ | GS | QNI | KCD |
| PDBID:3KU7 | | | | | | |
| AATA | RTL | RTL | LPYMEE | | | |
| PDBID:3Q1D | | | | | | |
| CEEHEEE | LSCE | GAHKDC | | | | |
| PDBID:3R3K | | | | | | |
| QG | | | | | | |
| PDBID:3T3J | | | | | | |
| KPYTFEDY | SG | GDL | PSS | TGK | SHDG | LSSLAY |
| PDBID:4DP2 | | | | | | |
| GAD-DGSLA | PS | PAG | SEED | AKG | CSPH | GM |
| PDBID:4DP4 | | | | | | |
| ADDG | PS | PAG | SEED | AKG | CSPH | GM |
| PDBID:4EC2 | | | | | | |
| SHG | PAF | PPNKQ | PLS | LNGE | LRNG | |
| PDBID:4GBN | | | | | | |
| TSI | | | | | | |
| PDBID:4IDL | | | | | | |
| QTGG | APGK | TTG | GDFVKGR | NANN | DSL | DGAR |
| PDBID:4JJD | | | | | | |
| DPN | LYD | ATLS | TKG | NHNGE | KN | |
| PDBID:4JZ3 | | | | | | |

| | | | | | | |
|---|---|---|---|---|---|---|
| DASVL | RPNNAVF | LPPK | KRGT | SVDEAVT | PPFS | LGKM |
| PDBID:3LHC | | | | | | |
| GS | RTNG | LNSVI | DG | GSS | TAAG | DG |
| PDBID:3NZ3 | | | | | | |
| KVTN | KVTG | IDK | DIP | SDS | | |
| PDBID:3QD7 | | | | | | |
| TGFL | PLSQ | REGL | LLRQ | DDKS | FDD | |
| PDBID:3RSD | | | | | | |
| DSST | NLT-KDR | CKNG | TGSS | YP | GNPY | |
| PDBID:3T8T | | | | | | |
| KEGQ | RGAKG | RVP | IESE | | | |
| PDBID:3VXW | | | | | | |
| FKN | AEKS | IDKR | ADL | PPEKA | ND | PTAA |
| DKDG | | | | | | |
| PDBID:4BS7 | | | | | | |
| LDN | NF | TQA | NTDG | GILQ | SRWW | DGRTPGS |
| NLCN | SSD | DGNGMNAW | KGTD | RGC | | |
| PDBID:4E1R | | | | | | |
| DDFDG | DG | DDFDG | DG | | | |
| PDBID:4EES | | | | | | |
| DPRLPDN | ILG | GPET | TKSG | DQKG | | |
| PDBID:4ETD | | | | | | |
| LDN | NF | TQA | NTDG | GILQ | SRW | DGRTPGS |
| LCN | SSD | DGNGMNAW | KGTD | RGC | | |
| PDBID:4HE7 | | | | | | |
| ENY | LAN | DEKR | YCE | | | |
| PDBID:4HV2 | | | | | | |
| LDN | NF | TQA | NTDG | GILQ | SRW | DGRTPGS |
| LCN | SSD | KGT | RGC | | | |
| PDBID:4IC9 | | | | | | |

| | | | | | | |
|---|---|---|---|---|---|---|
| TETD | KKG | NTEG | SLTTG | | | |
| PDBID:4KV4 | | | | | | |
| DVEAL-GLH | IKHP | PPDH | | | | |
| PDBID:4MDQ | | | | | | |
| DEKQQH | CSND | VKE | | | | |
| PDBID:4QYW | | | | | | |
| PD | MPEMN | DPNA | MG | PF | | |
| PDBID:4XO2 | | | | | | |
| MT | MT | | | | | |
| PDBID:5C4P | | | | | | |
| PD | PG | AEPP | GAGE | NQD | GPGG | |
| PDBID:5DKN | | | | | | |
| EGDKH | SHFL | DSDG | FFE | | | |
| PDBID:5EH4 | | | | | | |
| EPE | | | | | | |
| PDBID:5HDG | | | | | | |
| DAL | SPSG | FD | PC | RG | | |
| PDBID:5NWX | | | | | | |
| TTG | RTG | GR | LNDG | YP | | |
| PDBID:5OC8 | | | | | | |
| EK | CSND | VKE | | | | |
| PDBID:5UEV | | | | | | |
| DVEALGL | IKHP | PPDH | | | | |
| PDBID:5UVY | | | | | | |
| DVEAL-GLH | IKHP | PPDH | | | | |
| PDBID:6EVL | | | | | | |
| DPSH | | | | | | |
| PDBID:6PAZ | | | | | | |
| AE | PA | NPG | VDK | IKDM | PEGA | KINE |
| CTIH | GDSP | | | | | |
| PDBID:1B0T | | | | | | |
| IKCK | CPVD | PN | HPDEC | DGVKG | | |

| | | | | | | |
|---|---|---|---|---|---|---|
| VGVGGKS | EPGD | GLLN | GLDT | | | |
| PDBID:4IPF | | | | | | |
| DEKQQH | CSND | FGVQ | VKE | | | |
| PDBID:4MZ2 | | | | | | |
| VNGT | SAFGF | SSDA | EKS | DDR | SMNN | RDL |
| PDBID:4N5T | | | | | | |
| GEGT | EEV | DKQRQH | CHDD | VK | WAQSA | |
| PDBID:4P15 | | | | | | |
| GEG | HSY | | | | | |
| PDBID:4P7U | | | | | | |
| CEKPQE | DEN | DPKL | HD | LE-DAAS | PG | SDE |
| PDBID:4P9E | | | | | | |
| CKRM | NG | PSGF | NPFV | IDGS | | |
| PDBID:4PE7 | | | | | | |
| EGDKH | SHFL | DSDG | | | | |
| PDBID:4RLN | | | | | | |
| LDN | NF | TQA | NTDG | GILQ | SRW | DGRTPGS |
| LCN | SSD | DGNGMNAW | KGTD | RG | GC | CR |
| PDBID:4RUD | | | | | | |
| SSRTE | PEGE | HG | SKC | GAYN | TDLC | SRTE |
| PEGE | HG | SKC | GAYN | TD | DL | LC |
| CN | NK | | | | | |
| PDBID:4WO6 | | | | | | |
| LDN | NF | TQA | NTDG | GILQ | SRW | DGRTPGS |
| LCN | SSD | DGNGMNAW | KGT | RGC | | |
| PDBID:5CPV | | | | | | |
| FAGVLN | AADS | GLTSK | DQDK | FKADA | DSDG | |
| PDBID:5E0Z | | | | | | |
| VAG | PAG | PPAG | VDS | LSG | HN | PPAG |
| RDG | | | | | | |
| PDBID:5E4P | | | | | | |
| LD | RG | NF | TQA | NTDG | GILQ | SRWWC |

| | | | | | | | | | | | | | |
|---|---|---|---|---|---|---|---|---|---|---|---|---|---|
| | | | | K | | | | | | | | | |
| PDBID:1BFE | | | | | | | DGRTPGS | NLCN | SSD | NGMNAW | CKGTD | IRGC | |
| RGST | GEDGE | LAGG | RKG | NG | LRNA | GQ | PDBID:5EN9 | | | | | | |
| EAN | NSSG | | | | | | TSI | | | | | | |
| PDBID:1C0B | | | | | | | PDBID:5G25 | | | | | | |
| KET | DSST | NLT-KDRC | CKNGQTN | KYPN | GNPY | | GG | AQDG | CPTAGD | EE | | | |
| PDBID:1C76 | | | | | | | PDBID:5UEP | | | | | | |
| DGKG | KPG | TAYKE | DPSA | KNKK | TEKG | LSE-HIKNP | DVEALGL | IKHP | PPDH | | | | |
| PDBID:1CUO | | | | | | | PDBID:5UVZ | | | | | | |
| STR | PASCA | PKTGMG | GGG | SKDE | YPGHFSMM | | DVEAL-GLH | IKHP | PPDH | | | | |
| PDBID:1DDV | | | | | | | PDBID:5VR3 | | | | | | |
| DPNTK | STRN | GS | TPNM | SQK | | | PAIP | EHNP | | | | | |
| PDBID:1E5B | | | | | | | PDBID:5XWE | | | | | | |
| WSD | SSA | NGSQ | SGST | NGSGN | KNGS | | IDSC | PEGE | YT | PKTE | YT | IDSC | PEGE |
| PDBID:1EFQ | | | | | | | WKI | PKTE | | | | | |
| PD | SLG | SQS | SSNS | KPGQ | WAS | ESGVPDR | PDBID:6FGT | | | | | | |
| GT | SS | YYSTPY | | | | | SMSV | HED-AWPFL | NLKLVPG | IKKP | EDDS | | |
| PDBID:1EN2 | | | | | | | PDBID:6H0L | | | | | | |
| SQGG | IWG | CGRT | RSDH | GN | GQD | VHG | LDN | NF | TQA | NTDG | GILQ | SRW | DGRTPGS |
| PDBID:1EOT | | | | | | | LCN | SSD | DGNGMNAW | KGTD | RGC | | |
| PASVP | CFNL | PLQR | SGKCPQK | KLA | DPKK | | PDBID:1A18 | | | | | | |
| PDBID:1FAO | | | | | | | CDAFV | GD | STFK | TADD | GG | DG | GD |
| LG | GGLVK | RN | KDQMSPE | YSQERV | PF | | KG | | | | | | |
| PDBID:1FAZ | | | | | | | PDBID:1B6E | | | | | | |
| DLCTQA | NPFGFP | | | | | | QEK | RC | WENG | SQYLFPS | NTK | NPNG | CED |
| PDBID:1FDA | | | | | | | PDBID:1C9V | | | | | | |
| IKCK | CPVD | PN | HPDEC | CPAQ | PEDM | LPD | DSST | NLT-KDR | CKNG | TGSS | YP | GNPY | |
| WDGVKGK | | | | | | | PDBID:1CS3 | | | | | | |
| PDBID:1FE3 | | | | | | | GTLC | DSQ | SQ | DFL | | | |
| VEAFC | GD | STFK | LG | TADD | DGDK | DG | PDBID:1DCF | | | | | | |

| | | | | | | |
|---|---|---|---|---|---|---|
| DG | GD | | | | | |
| PDBID:1FER | | | | | | |
| IKCK | CPVD | PN | HPDEC | EDEVPEDM | DGVKGK | |
| PDBID:1FEV | | | | | | |
| NLTKDR | CKNG | TGSS | YP | GNPY | | |
| PDBID:1FOW | | | | | | |
| MTFITKT | IESG | RSMG | | | | |
| PDBID:1GD6 | | | | | | |
| SR | TSK | NRNG | GLFQ | DRYW | KDCN | TDD |
| RFDAW | HCQGS | | | | | |
| PDBID:1GHJ | | | | | | |
| PESIA | WHKKPGE | KRD | DKV | NEGD | LSG | |
| PDBID:1GKH | | | | | | |
| AQ | SRQG | NEYP | DEGQ | GQFG | DRL | |
| PDBID:1GV2 | | | | | | |
| GPKR | LKGR | GNR | LPGR | | | |
| PDBID:1GXT | | | | | | |
| VQGV | DGDG | PPLA | SQL | | | |
| PDBID:1H2P | | | | | | |
| RN | VPGG | FG | NTG | ST | ISGSS | IDN |
| QGERDH | YR | NKGF | EH | NNDE | | |
| PDBID:1IDI | | | | | | |
| TAT | NLCY | CDAF | SRGK | KPYE | TDKC | |
| PDBID:1J7Z | | | | | | |
| NLTKDR | CKNG | TGSS | YP | GNPY | | |
| PDBID:1JPO | | | | | | |
| LDN | NF | TQA | NTDG | GILQ | SRWW | DGRTPGS |
| NLCN | SSD | KGTD | RGC | | | |
| PDBID:1KM8 | | | | | | |
| DN | GG | TGV | PRPC | NQ | GR | |
| PDBID:1KSM | | | | | | |
| KEGDPN | DKNG | | | | | |

| | | | | | | |
|---|---|---|---|---|---|---|
| FTGL | SHEHK | VEN | KPV | | | |
| PDBID:1EMN | | | | | | |
| DG | PFG | GN | NPCGNG | IG | TCEEGF | GPMM |
| PDBID:1EOQ | | | | | | |
| GPSE | STL | | | | | |
| PDBID:1EOW | | | | | | |
| DSST | NLT-KDR | CKNG | TGSS | YP | GNPY | |
| PDBID:1EQV | | | | | | |
| GD | KG | LL | ETK | PKK | AKK | DKYG |
| DG | AP | | | | | |
| PDBID:1FIK | | | | | | |
| YKDSP | VPGK | GG | LLQDGEF | TGGA | TDK | GV |
| PDBID:1FKD | | | | | | |
| DGRT | KRG | EDG | GK | SVG | ATG | PGI |
| PPH | | | | | | |
| PDBID:1FKF | | | | | | |
| DGRT | KRG | EDG | GK | SVG | ATG | PGI |
| PPH | | | | | | |
| PDBID:1HEY | | | | | | |
| DKEL | TGKS | NN | WNMPNMD | DGAMSAL | KPF | |
| PDBID:1HME | | | | | | |
| DPNA | HPGL | | | | | |
| PDBID:1IOS | | | | | | |
| LDN | NF | TQA | NTDG | GILQ | SRWW | DGRTPGS |
| NLCN | SSD | DGN | KGTD | RGC | | |
| PDBID:1JPE | | | | | | |
| QH | EFY | DAG | | | | |
| PDBID:1KM9 | | | | | | |
| DN | GG | TGVI | PRPC | NQ | GR | |
| PDBID:1KXX | | | | | | |
| LNN | NF | TQA | NTDG | GILQ | SRWW | DGRTPGS |
| NLCN | SSD | DGN | KGTD | RGC | | |

| | | | | | | |
|---|---|---|---|---|---|---|
| QL | | | | | | |
| PDBID:1KTH | | | | | | |
| PNTK | NENK | | | | | |
| PDBID:1LOZ | | | | | | |
| MDG | GY | TRA | AGDR | GTFQ | SRYW | DGKTPGA |
| NACH | QDN | DPQ | QNR | VQGC | | |
| PDBID:1MH7 | | | | | | |
| VPAR | GCY | GG | QG | KGRN | NI | |
| PDBID:1O4K | | | | | | |
| EEW | KI | NAENPRG | SETTKGA | NAK | LDSG | SR |
| ADGLCHR | | | | | | |
| PDBID:1O4L | | | | | | |
| EEW | KI | NAENPRG | SETTKG | NAK | LDSG | SR |
| ADGLCHR | | | | | | |
| PDBID:1O4Q | | | | | | |
| EEW | KI | NAENPRG | SETTKG | NAK | LDSG | SR |
| ADGLCHR | | | | | | |
| PDBID:1RTU | | | | | | |
| PQ | GG | YP | SEDI | VYNG | RD | TNTG |
| HTGA | SYDG | | | | | |
| PDBID:1RZY | | | | | | |
| KEIPA | DDQ | DISP | AP | KKH | LKKG | QSVY |
| NWPP | | | | | | |
| PDBID:1TUW | | | | | | |
| HD | RSH | AQDW | | | | |
| PDBID:1U3J | | | | | | |
| DRT | VTG | KD | EV | RQF | YKDVNI | KSDL |
| PDBID:1UIC | | | | | | |
| LDN | NF | TQA | NTDG | GILQ | SRWW | DGRTPGS |
| NLCN | SSD | DGN | KGT | RGC | | |

| | | | | | | |
|---|---|---|---|---|---|---|
| PDBID:1LXI | | | | | | |
| WQDW | AFPL | NPETV | DDSS | | | |
| PDBID:1MG6 | | | | | | |
| GCNC | NK | HL | | | | |
| PDBID:1O2E | | | | | | |
| IPSS | NN | GCY | NPYTN | NN | SSEN | NLDMKN |
| PDBID:1O45 | | | | | | |
| EEW | KI | NAENPRG | SETTKG | NAK | LDSG | SR |
| ADGLCHR | | | | | | |
| PDBID:1O4G | | | | | | |
| SIQAEEW | KI | NAENPRG | SETTKGA | NAK | LDSG | SR |
| ADGLCHR | | | | | | |
| PDBID:1Q8B | | | | | | |
| DLNEI | EEG | | | | | |
| PDBID:1R26 | | | | | | |
| MRAR | AVWC | FPTV | ADNN | QLP | SG | GAN |
| PDBID:1RLK | | | | | | |
| KDLD | GYTQVE | | | | | |
| PDBID:1SDZ | | | | | | |
| FTDW | LDWL | GD | FFCG | EQED | PIN | |
| PDBID:1SNQ | | | | | | |
| GD | KG | LL | ETVEKY | AKK | DKYG | DG |
| KG | | | | | | |
| PDBID:1SSC | | | | | | |
| DSST | NLT-KDR | CKNG | TGSS | YP | | |
| PDBID:1TAY | | | | | | |
| MDG | GY | TRA | AGDR | GIFQ | SRAW | DGKTPGA |
| NACH | QDN | DPQ | QNR | VQGC | | |
| PDBID:1TXA | | | | | | |

| | | | | | | |
|---|---|---|---|---|---|---|
| PDBID:1VHF | | | | | | |
| RLIA | KG | YETP | LTEY | | | |
| PDBID:1ZIA | | | | | | |
| KD | PA | APG | PTDK | IKGM | PDGA | KINE |
| CTPH | GDAP | | | | | |
| PDBID:1ZJ7 | | | | | | |
| LWQRP | GG | DTGA | IG | CG | | |
| PDBID:2B8G | | | | | | |
| KAG | EKG | MK | DRS | KEG | NEG | SNST |
| PDBID:2BRF | | | | | | |
| PPGE | PSDGQ | PLTQ | DRK-CSRTQ | PETR | GQ | KPG |
| VG | NG | | | | | |
| PDBID:2BZY | | | | | | |
| DKT | EVG | NING | NG | DKT | EVG | NING |
| NG | PQ | QN | | | | |
| PDBID:2CW4 | | | | | | |
| TDRAP | AQ | GG | IP | APDG | DE | TPPY |
| PDBID:2HB4 | | | | | | |
| LWKRP | GG | DTGA | IG | CG | GPTPVN | |
| PDBID:2J5A | | | | | | |
| KPTL | QK | DED | | | | |
| PDBID:2JK7 | | | | | | |
| PRN | FGTW | GD | FHCG | KPSE | YPGC | |
| PDBID:2NUH | | | | | | |
| RL | WQGK | YRLP | | | | |
| PDBID:2OPY | | | | | | |
| PRN | GTW | GD | FHCG | KPSE | PGC | |
| PDBID:2PPI | | | | | | |
| DG | EGI | | | | | |
| PDBID:2V6H | | | | | | |

| | | | | | | |
|---|---|---|---|---|---|---|
| PDAT | KPGV | DNC | TWKRK | | | |
| PDBID:1U68 | | | | | | |
| YNR | ENP | | | | | |
| PDBID:1U9R | | | | | | |
| GD | KG | LL | ETVEKY | AKK | DKYG | DG |
| KG | | | | | | |
| PDBID:1UID | | | | | | |
| LDN | NF | TQA | NTDG | GILQ | SRWW | DGRTPGS |
| NLCN | SSD | DGNGMNAW | KGTD | RGC | | |
| PDBID:1YEB | | | | | | |
| QQCH | EEGG | NKVGP | LHG | SGQVKGY | TN | IPGT |
| PDBID:208L | | | | | | |
| MDG | GY | TRA | AGDR | GIFQ | SRYW | DGKTPGA |
| NAAH | QDN | DPQ | QNR | QGC | | |
| PDBID:2D4M | | | | | | |
| ATPGS | PPN | DNDY | VNN | SQG | | |
| PDBID:2FO3 | | | | | | |
| PIN | HPNNIR | LEN-TIYAN | PDDY-PLKP | QKP | TH | YSNG |
| GDDYNPSL | | | | | | |
| PDBID:2FTB | | | | | | |
| NGD | PN | IG | SMGG | GG | TDQ | GN |
| GG | | | | | | |
| PDBID:2H8A | | | | | | |
| NKV | NDL | VPF | SLSG | QPNR | | |
| PDBID:2JP8 | | | | | | |
| RVYI | | | | | | |
| PDBID:2MSS | | | | | | |
| SVNT | VD | DKTTNRHR | FES | NN | KA | |
| PDBID:2OQK | | | | | | |
| EEG | NG | FDG | NPG | DF | GEIPETT | |

| | | | | | | |
|---|---|---|---|---|---|---|
| TVGG | GKW | QH | RASK | TDA | TKDK | |
| PDBID:3A0V | | | | | | |
| KDG | VLG | LPD | KGER | NAKTQ | | |
| PDBID:3BI9 | | | | | | |
| LGQ | PNSKCNA | DGTR | STK | KVQFG | SNT | GWFN |
| AL | LV | | | | | |
| PDBID:3ENU | | | | | | |
| KNF | PRL | PFG | WENK | GPR | HNY | DAGA |
| ANL | FDNF | | | | | |
| PDBID:3EZM | | | | | | |
| GS | RTNG | LNSV | DG | NFIET | GSS | TRAQ |
| DG | | | | | | |
| PDBID:3FAJ | | | | | | |
| KKGD | | | | | | |
| PDBID:3GLW | | | | | | |
| RNF | TR | GQ | | | | |
| PDBID:3GM3 | | | | | | |
| RTDD | AQL | AVT | | | | |
| PDBID:3GQU | | | | | | |
| PSS | LGAHG | PWW | IAE | | | |
| PDBID:3I3Z | | | | | | |
| TSI | FVNQ | | | | | |
| PDBID:3I7W | | | | | | |
| DSSTS | NLT-KDR | KP | CKNG | TGSS | YP | GNPY |
| PDBID:3I7X | | | | | | |
| DSSTS | NLT-KDR | KP | CKNG | TGSS | YP | GNPY |
| PDBID:3IE9 | | | | | | |
| ADGA | KM | ETP | KVG | VAGVLG | KKE | TP |
| PDBID:3INC | | | | | | |
| TSI | | | | | | |
| PDBID:3J4G | | | | | | |
| LDNYRGY | NF | TQA | NTDGS | GILQ | SRWW | TPGS |
| NLCN | SSD | KGT | IRGC | | | |

| | | | | | | |
|---|---|---|---|---|---|---|
| PDBID:2OUB | | | | | | |
| SS | GCY | GWG | NG | LY | | |
| PDBID:2P61 | | | | | | |
| SPT | KL | EWQTI | | | | |
| PDBID:2PNE | | | | | | |
| CPG | GPG | NPGC | GT | GTPK | | |
| PDBID:2PVT | | | | | | |
| GCY | GG | NG | LY | | | |
| PDBID:2QDB | | | | | | |
| GN | KGQ | LL | AKK | DKYG | DG | KG |
| PDBID:2QHW | | | | | | |
| GCY | WG | NG | LY | | | |
| PDBID:2REA | | | | | | |
| DGR | YNPD | EGQ | RMVLGRT | | | |
| PDBID:2REY | | | | | | |
| MEYN | MKGG | EPG | ND | FENM | | |
| PDBID:2UUX | | | | | | |
| PIGW | MGNC | | | | | |
| PDBID:2VSL | | | | | | |
| PRN | FGTW | GD | FHCG | KPSE | PGC | |
| PDBID:2W51 | | | | | | |
| RPGDC | TFS | TDDA | DSQICEL | KKL | GETCKGC | |
| PDBID:3BKS | | | | | | |
| DRSKR | EG | FNDL | ELAV | TADYP | KETS | |
| PDBID:3CQ1 | | | | | | |
| DPELG | VVNLG | PP | PLHD | LPGV | FEPP | RLL |
| PDBID:3DML | | | | | | |
| QPGC | QRD | PPGL | LARP | FTP | GD | |
| PDBID:3G7C | | | | | | |
| WIREYP | REES | | | | | |
| PDBID:3KLU | | | | | | |
| LSFFPGQT | PISKRF | DKEG | TTEL | PTFKA | | |
| PDBID:3L1 | | | | | | |

| | | | | | | |
|---|---|---|---|---|---|---|
| PDBID:3JZR | | | | | | |
| KDT | DEKQQH | CSND | VKE | | | |
| PDBID:3KVT | | | | | | |
| GG | KIPAT | TEGMLN | PVLN | PTDVC | | |
| PDBID:3ONH | | | | | | |
| PQD | ASNQ | YD | EDLNDR | GNG | EEGDT | DELPCNT |
| PDBID:3S9K | | | | | | |
| NLETYEW | SI | KEG | SRTPGT | KAIISEN | DSPK | FDS |
| GGGLVTR | SPGI | | | | | |
| PDBID:3UAF | | | | | | |
| CPTD | KK | DRNG | GP | HRDSN | PS | LDR |
| PDBID:3WVT | | | | | | |
| PQCKEE | GT | SPG | GELT | APR | LSKC | ST |
| DQG | GD | | | | | |
| PDBID:4DP0 | | | | | | |
| ADDGSLA | PS | PAG | SEED | AKG | CSPH | GM |
| PDBID:4F68 | | | | | | |
| FGFKGV | | | | | | |
| PDBID:4FE6 | | | | | | |
| LWQRP | GG | DTGA | IG | CG | PTPVN | |
| PDBID:4GQV | | | | | | |
| KPTT | DEDW | EKT | DSDG | | | |
| PDBID:4K59 | | | | | | |
| EGE | KADY | GN | PRG | | | |
| PDBID:4KGT | | | | | | |
| GG | DG | GGL | DG | | | |
| PDBID:4LFQ | | | | | | |
| RKTCG | | | | | | |
| PDBID:4LTT | | | | | | |
| KPF | PD | DD | | | | |

| | | | | | | |
|---|---|---|---|---|---|---|
| M | | | | | | |
| GK | | | | | | |
| PDBID:3LLH | | | | | | |
| EPN | GD | GQP | GD | PS | | |
| PDBID:3O70 | | | | | | |
| LYFQGL | FAGR | NECH | KS | VPEV | | |
| PDBID:3P2X | | | | | | |
| CTS | | | | | | |
| PDBID:3PAZ | | | | | | |
| AE | PA | NPG | VDK | IKDM | PEGA | KINE |
| CTPH | GDSP | | | | | |
| PDBID:3RNT | | | | | | |
| CD | GS | GSNS | NYEGFDF | LSSG | ENN | HTGASGNN |
| PDBID:3UB2 | | | | | | |
| SRWSKD | GAI | QALS | APGAE | SGL | GRG | |
| PDBID:3UB3 | | | | | | |
| SRWSKD | SEE | GAI | QALS | PGAEG | SGL | GRG |
| PDBID:3UMD | | | | | | |
| EHVAFGS | GDG | VIG | DSPEF | DYQM | SGD | |
| PDBID:3UME | | | | | | |
| AFGS | GDG | VIG | DYQM | SGD | | |
| PDBID:3V1G | | | | | | |
| TSI | GRG | TSI | GRG | | | |
| PDBID:3WUN | | | | | | |
| LDN | NF | TQA | NTDG | GILQ | SRW | DGRTPGS |
| LCN | SSD | DGNGMNAW | KGT | IRGC | | |
| PDBID:4AYA | | | | | | |
| VPSIPQNK | HLKPSF | VPSIPQNK | | | | |
| PDBID:4CGQ | | | | | | |
| QPQN | AEHV | RMEE | | | | |
| PDBID:4DH4 | | | | | | |

| | | | | | | |
|---|---|---|---|---|---|---|
| PDBID:4NEJ | | | | | | |
| KKA | | | | | | |
| PDBID:4OXW | | | | | | |
| EGKSK | GG | PELV | IYSAL | | | |
| PDBID:4TQN | | | | | | |
| VKNP | YQE | RKTS | | | | |
| PDBID:5B79 | | | | | | |
| CDFCL | SKINKKTG | CSDCG | KTYRW | CIECK | NICG | SEN |
| CDDCD | LTPSMSEP | ASIY | | | | |
| PDBID:5BPO | | | | | | |
| TSI | TSI | | | | | |
| PDBID:5PAL | | | | | | |
| DPGT | LKGK | DKDQ | SAHG | DSDH | | |
| PDBID:5UVS | | | | | | |
| DVEALGL | IKHP | PPDH | | | | |
| PDBID:6BBK | | | | | | |
| SATPKDT | STL | NQDG | KNDG | | | |
| PDBID:6CEC | | | | | | |
| CPH | PAAG | DVTQ | CGDCG | IQE | LSCY | GRYING |
| YIDL | YYCQ | | | | | |
| PDBID:6CEF | | | | | | |
| PWCPH | PAAG | DVTQ | CGDCG | IQE | LSCY | GRYING |
| YIDL | YYCQ | | | | | |
| PDBID:6EVM | | | | | | |
| DPSH | | | | | | |
| PDBID:6FH6 | | | | | | |
| SMSV | HED-AWPFL | NLKLVPG | IKKP | EDDS | | |
| PDBID:6IFH | | | | | | |
| MKIPGM | TPDV | AYGE | KPF | | | |

| | | | | | | |
|---|---|---|---|---|---|---|
| VAA | GG | GG | GD | | | |
| PDBID:4ETA | | | | | | |
| LDN | NF | TQA | NTDG | GILQ | SRW | DGRTPGS |
| LCN | SSD | DGNGMNAW | KGTD | RGC | | |
| PDBID:4ETB | | | | | | |
| LDN | NF | TQA | NTDG | GILQ | SRW | DGRTPGS |
| LCN | SSD | DGNGMNAW | KGT | RGC | | |
| PDBID:4ETE | | | | | | |
| LDN | NF | TQA | NTDG | GILQ | SRW | DGRTPGS |
| LCN | SSD | DGNGMNAW | KGTD | RGC | | |
| PDBID:4EWW | | | | | | |
| TSI | | | | | | |
| PDBID:4EWZ | | | | | | |
| TSI | | | | | | |
| PDBID:4F6D | | | | | | |
| VN | FGFKGV | | | | | |
| PDBID:4GSP | | | | | | |
| CD | GS | GSNS | NYEG | LSSG | ENN | HTGASGNN |
| PDBID:4HB6 | | | | | | |
| KAKNG | VPD | GKGC | | | | |
| PDBID:4JHB | | | | | | |
| GS | NKE | CTG | KE | TASW | RQF | YEDLEI |
| RLTDG | | | | | | |
| PDBID:4LJN | | | | | | |
| SFCL | KEQNREK | CADCG | CIECK | SSCR | ADN | CDSCD |
| CDPP | PKGM | KK | KG | | | |
| PDBID:4P2P | | | | | | |
| IPGS | NN | GCY | LDSC | NT | NSKN | NLDTKKY |
| PDBID:4RL | | | | | | |

| | | | | | | |
|---|---|---|---|---|---|---|
| D | | | | | | |
| PDBID:1AB0 | | | | | | |
| GDAFV | GD | STHK | TADD | GG | DG | GD |
| KG | | | | | | |
| PDBID:1ACD | | | | | | |
| GDAFV | GD | STHK | TADD | GG | DG | GD |
| KG | | | | | | |
| PDBID:1B1J | | | | | | |
| QDNS | DAKPQGR | TSPC | ENKN | REN | WPPC | ENGL |
| QSIFRR | | | | | | |
| PDBID:1BM2 | | | | | | |
| HPW | KI | HDG | SESA-PGD | GN | DGAG | LWV |
| RNQ | | | | | | |
| PDBID:1BQK | | | | | | |
| KD | PA | APG | PTDK | IKGM | PDGA | KINE |
| CTPH | GDAP | | | | | |
| PDBID:1D1M | | | | | | |
| NADG | DG | NADG | DG | | | |
| PDBID:1DDW | | | | | | |
| DPNTK | STRN | GS | SQK | SRAN | | |
| PDBID:1E6L | | | | | | |
| DKEL | WNMPNMD | SAL | AEA | KPF | | |
| PDBID:1FDD | | | | | | |
| IKCK | EVCPVD | PN | HPDEC | PEDM | DGVKGK | |
| PDBID:1FE5 | | | | | | |
| GCY | EP | DTAD | MISSSTNC | | | |
| PDBID:1FF2 | | | | | | |
| IKCK | PN | HPDEC | PEDM | DGVKG | | |
| PDBID:1FLQ | | | | | | |
| LDN | NF | TQA | NTDG | GILQ | SRWW | DGRTP |

| | | | | | | |
|---|---|---|---|---|---|---|
| M | | | | | | |
| LDN | NF | TQA | NTDG | GILQ | SRW | DGRTPGS |
| LCN | SSD | DGNGMNAW | KGT | RG | GC | CR |
| PDBID:4YM8 | | | | | | |
| LDN | NF | TQA | NTDG | GILQ | SRW | DGRTPGS |
| LCN | SSD | NGMNAW | KGT | RGC | | |
| PDBID:4YOP | | | | | | |
| LDN | NF | TQA | NTDG | GILQ | SRW | DGRTPGS |
| LCN | SSD | DGNGMNAW | KGTD | RGC | | |
| PDBID:5CO9 | | | | | | |
| TSI | | | | | | |
| PDBID:5DM9 | | | | | | |
| LDN | NF | TQA | NTDG | GILQ | SRW | DGRTPGS |
| LCN | SSD | NGMNAW | KGTD | IRGC | | |
| PDBID:5HBP | | | | | | |
| IPE | HE | APA | ED | | | |
| PDBID:5HMB | | | | | | |
| PD | PHG | GDR | GAT | DAAG | | |
| PDBID:5HQI | | | | | | |
| TSI | FVNQ | | | | | |
| PDBID:5LAW | | | | | | |
| EKQQH | CSND | VKE | | | | |
| PDBID:5LN2 | | | | | | |
| SQIP | SVG | DEKQQH | CSND | VKE | | |
| PDBID:6CE6 | | | | | | |
| CPH | PAAG | DVTQ | CGDCG | IQE | LSCY | GRYING |
| YIDL | YYCQ | | | | | |
| PDBID:6DXH | | | | | | |
| SKHAFSL | CSKC | REN | LTDG | YFDG | PD | YDED |
| DMLKM | | | | | | |

| | | | | | | |
|---|---|---|---|---|---|---|
| | | | | | | GS |
| NLCN | SSD | DGNGMNAW | KATD | RGC | | |
| PDBID:1FLY | | | | | | |
| LDN | NF | TQA | NTDG | GILQ | SRWW | DGRTPGS |
| NLCN | SSD | DAN | KGTD | RGC | | |
| PDBID:1GOD | | | | | | |
| KDKT | | | | | | |
| PDBID:1HEH | | | | | | |
| VRG | WAD | SGSSSW | NGGQ | PNGS | KNGS | |
| PDBID:1HRC | | | | | | |
| AQCH | EKGG | HKTGP | LHGLFG | TGQAPGF | IPGT | |
| PDBID:1J73 | | | | | | |
| GERG | | | | | | |
| PDBID:1JZA | | | | | | |
| KSTG | AKNQG | YAF | GLPEST | PN | KSTG | AKNQG |
| YAF | GLPEST | PN | | | | |
| PDBID:1K1Z | | | | | | |
| AQDKKR | ELGL | GIPP | PGAF | NPG | AEAEH | TATN |
| PCNR | | | | | | |
| PDBID:1M1S | | | | | | |
| PP | PATG | SNNEH | PV | DAKG | PAEE | AP-FKAG |
| PDBID:1O41 | | | | | | |
| EEW | KI | NAENPRG | SETTKGA | NAK | LDSG | SR |
| ADGLCHR | | | | | | |
| PDBID:1O47 | | | | | | |
| EEW | KI | NAENPRG | SETTKGA | NAK | LDSG | SR |
| ADGLCHR | | | | | | |
| PDBID:1O4R | | | | | | |
| SIQAEEW | KI | NAENPRG | SETTKGA | NAK | LDSG | SR |
| ADGLCHR | | | | | | |
| PDBID:1O | | | | | | |

| | | | | | | |
|---|---|---|---|---|---|---|
| PDBID:6INS | | | | | | |
| GERG | TP | PKG | TSI | | | |
| PDBID:6JQ4 | | | | | | |
| DPEN | GKRK | | | | | |
| PDBID:1A0B | | | | | | |
| SPDL | | | | | | |
| PDBID:1AF5 | | | | | | |
| NKE | PNQSYKFK | TQR | GS | LKLKQ | AALN | |
| PDBID:1ALB | | | | | | |
| CDAFV | GD | STFK | LG | TADD | GG | DG |
| GD | KG | | | | | |
| PDBID:1B1I | | | | | | |
| DAKPQGR | TSPCKD | ENKN | REN | WPPC | NG | |
| PDBID:1BAS | | | | | | |
| KNGG | HPDG | EKS-DPHI | RG | VSAN | KEDG | TDEC |
| ESNN | RKYTSW | KRTG | GPGQKAI | | | |
| PDBID:1CYI | | | | | | |
| AACH | NSVMPEKT | LDGG | GA | WADRL | | |
| PDBID:1DKJ | | | | | | |
| LDN | NF | SQA | NTDG | GVLQ | SRWW | DGKTPGS |
| NLCN | SSD | KGT | RGC | | | |
| PDBID:1EV3 | | | | | | |
| TSI | TSI | | | | | |
| PDBID:1HE7 | | | | | | |
| GQPA | NG | TSF | AANE | QP | PF | NPFE |
| PDBID:1HQ8 | | | | | | |
| PNNW | RN | IYS | IPANG | EDG | SYNQL | IPKG |
| SSF | DCAN | | | | | |
| PDBID:1I0V | | | | | | |
| CD | GS | GSNS | NYEG | LSSG | ENN | HTGAS |

| | | | | | | |
|---|---|---|---|---|---|---|
| R5 | | | | | | |
| SALT | EDDSV | RYG | | | | |
| PDBID:1PAL | | | | | | |
| SFAG | AADS | DQDK | FSPSA | DKDG | | |
| PDBID:1Q4R | | | | | | |
| KDGV | HQGY | | | | | |
| PDBID:1QL2 | | | | | | |
| IDT | IDT | IDT | | | | |
| PDBID:1T00 | | | | | | |
| HMAG | TDD | LKN | AAWC | GDKI | IDEN | SIP |
| GG | | | | | | |
| PDBID:1UIA | | | | | | |
| DN | NF | TQA | NTDG | GILQ | SRWW | DGRTPGS |
| NLCN | SSD | GN | KGT | RGC | | |
| PDBID:1UWM | | | | | | |
| EHNG | KPG | AYEP-NPAT | DG | | | |
| PDBID:1WAQ | | | | | | |
| WDDW | PL | EFPL | DPEST | DSAN | | |
| PDBID:1WHI | | | | | | |
| QE | GSGR | NIG | TKRG | RPDG | RDDK | AP |
| PDBID:1WY9 | | | | | | |
| SND | NGNG | SEE | | | | |
| PDBID:1YGT | | | | | | |
| GGN | KP | NNDTD | NKT | | | |
| PDBID:1Z21 | | | | | | |
| SNPP | | | | | | |
| PDBID:2A9I | | | | | | |
| TPST | PQE | KPSG | RYN | LQTGL | | |
| PDBID:2AZ8 | | | | | | |
| LWKRP | GG | DTGA | LPG | IG | CG | GPT |
| PDBID:2BQQ | | | | | | |

| | | | | | | |
|---|---|---|---|---|---|---|
| | | | | | | GNN |
| PDBID:1I3Z | | | | | | |
| LPY | GCL | VDG | ESVPG | KK | EKHGY | DAHT |
| PN | | | | | | |
| PDBID:1IOR | | | | | | |
| LDN | NF | TQA | NTDG | GIFQ | SRWW | DGRTPGS |
| NLCN | SSD | DGN | KGTD | RGC | | |
| PDBID:1IRW | | | | | | |
| EKGG | HKVGP | LHGIFG | SGQAEGY | IPGT | | |
| PDBID:1JDL | | | | | | |
| CMACH | GPDA | NLVGP | LTGVID | AG-TAPGF | PD | |
| PDBID:1JKD | | | | | | |
| MDG | GY | TRA | AGDR | GIFQ | SRYW | DGKTPGA |
| NACH | QDN | DPQ | QNR | VQGC | | |
| PDBID:1KH0 | | | | | | |
| FANG | NG | FANG | NG | | | |
| PDBID:1KH8 | | | | | | |
| DSSTS | NLT-KDR | CKNG | TGSS | YP | | |
| PDBID:1KXI | | | | | | |
| LPFI | PEGK | KKFPL | DNC | SAL | TDKC | LPFI |
| PEGK | KKF | DNC | SAL | TDKC | | |
| PDBID:1L5D | | | | | | |
| LDVR | GPGGK | GPS | DE | GT | NYLP | GSG |
| AAAG | SPDG | DP | PA | AC | CD | DP |
| PE | EA | FS | AF | | | |
| PDBID:1LVE | | | | | | |
| PD | SLG | SQS | SSNS | KPGQ | WAS | ESGVPDR |
| GT | SS | QAEDV | STPY | | | |
| PDBID:1MB3 | | | | | | |
| LPEI | DDDLAHI | KPI | | | | |
| PDBID:1NEH | | | | | | |

| | | | | | | |
|---|---|---|---|---|---|---|
| NG | RYF | SSDRFR | NI | LPQG | IDG | EEGE |
| DDV | NPNW | | | | | |
| PDBID:2COQ | | | | | | |
| PR | ETGE | DTA | LGS | GR | RDL | AFN |
| PDBID:2EH9 | | | | | | |
| ELG | GK | GEEP | | | | |
| PDBID:2HWV | | | | | | |
| GD | AY | RG | IG | FGD | ED | PS |
| PTY | RGV | | | | | |
| PDBID:2JIN | | | | | | |
| DYLV | PS | LG | KENG | QEG | NG | LKNL |
| GY | | | | | | |
| PDBID:2O0P | | | | | | |
| RGQAN | AEPGED | DADG | | | | |
| PDBID:2O0Q | | | | | | |
| RGQAN | GED | DADG | | | | |
| PDBID:2OAI | | | | | | |
| REDG | DGTL | NNYH | LAG | HVG | AG | GA |
| PDBID:2OJR | | | | | | |
| DTNN | EGDE | DTNN | EGDELLA | TLTG | EPSD | AG |
| EDGR | LSDYN | QKES | LR | RG | | |
| PDBID:2ON8 | | | | | | |
| GKTL | | | | | | |
| PDBID:2P1X | | | | | | |
| GG | ENG | KQG | GD | DRSG | | |
| PDBID:2Q1M | | | | | | |
| GPLPSK | SEPP | DW | NANY | NK | NKSK | HVG |
| NSEHQ | | | | | | |
| PDBID:2X44 | | | | | | |
| PAVV | SSRG | AT | DS | MMGN | LDDS | SGN |
| PDBID:2YHN | | | | | | |

| | | | | | | |
|---|---|---|---|---|---|---|
| PAN | AADD | QD | QF | QLF-PGK | VNG | CASW |
| PDBID:1O3X | | | | | | |
| NKLI | SQG | TEDN | | | | |
| PDBID:1O5J | | | | | | |
| KG | YETP | LTEY | | | | |
| PDBID:1PZC | | | | | | |
| AE | PA | NPG | VDK | IKDM | PEGA | KINE |
| CTPH | GDSP | | | | | |
| PDBID:1REX | | | | | | |
| MDG | GY | TRA | AGDR | GIFQ | SRYW | DGKTPGA |
| NACH | QDN | DPQ | QNR | VQGC | | |
| PDBID:1RFP | | | | | | |
| LDN | NF | TQA | NTDG | GILQ | SRWW | DGRTPGS |
| NLCN | SSD | DGN | KGTD | RGC | | |
| PDBID:1RS2 | | | | | | |
| NVIDT | YNR | ENP | | | | |
| PDBID:1SNP | | | | | | |
| GD | KG | LL | ETVEKY | AKK | DKYG | DG |
| KG | | | | | | |
| PDBID:1T2J | | | | | | |
| QPGG | APGK | SGSG | AD-SVKGR | NSL | DWYGMD | |
| PDBID:1TCY | | | | | | |
| MDG | GY | TRA | AGDR | GIFQ | SRFW | DGKTPGA |
| NACH | QDN | DPQ | QNR | VQGC | | |
| PDBID:1TVQ | | | | | | |
| GD | PR | LG | TMDG | NG | EK | GN |
| GG | | | | | | |
| PDBID:1UW3 | | | | | | |
| LGGY | FGN | | | | | |
| PDBID:1VED | | | | | | |
| LDN | NF | TQA | NTDG | GILQ | SRWW | DGRTPGS |

| | | | | | | |
|---|---|---|---|---|---|---|
| CMVCCEE | PCG | CPVCR | CMVCC | PCG | CPVCR | |
| PDBID:2Z44 | | | | | | |
| PIQE | LPE | | | | | |
| PDBID:2ZHH | | | | | | |
| NSGNQ | SRSDC | | | | | |
| PDBID:3A7L | | | | | | |
| PAEL | SKEH | EADG | EVG | SAG | VKA | PVS |
| DSP | EPYAGG | SD | | | | |
| PDBID:3BOV | | | | | | |
| APKE | VG | ELE | SLQSERA | PS | GA | |
| PDBID:3BY5 | | | | | | |
| RKGA | KADE | TFS-QASL | GAGA | GD | | |
| PDBID:3DP5 | | | | | | |
| ASWS | AV | NTVHPEKT | RNPGPGM | GEAMIP | | |
| PDBID:3EAZ | | | | | | |
| MPW | KI | YPPETG | TNYPG | DG | AS | IDEEV |
| ADGLCTR | | | | | | |
| PDBID:3ERS | | | | | | |
| HE-NADKL | VGQK | LVPYYS | MG | CNL | RG | TDDG |
| PERM | PAG | | | | | |
| PDBID:3GBQ | | | | | | |
| ADD | KRG | QN | NG | | | |
| PDBID:3GK2 | | | | | | |
| EGDKH | SHFL | DSDG | | | | |
| PDBID:3GK4 | | | | | | |
| EGDKH | SHFL | DSDG | | | | |
| PDBID:3GSP | | | | | | |
| CD | GS | GSNS | NYEG | LSSG | ENN | HTGASGNN |
| PDBID:3HJX | | | | | | |
| RPMDEYS | TT | | | | | |
| PDBID:3M4T | | | | | | |

| | | | | | | |
|---|---|---|---|---|---|---|
| NLCN | SSD | NGMNAW | KGT | IRGC | | |
| PDBID:1Z3L | | | | | | |
| TAAA | NLT-KDR | CKNG | TGSS | YP | GNPY | |
| PDBID:2A9Q | | | | | | |
| PD | KPF | | | | | |
| PDBID:2APB | | | | | | |
| AA | PR | VTG | DTGH | AG | GDIPDG | QE |
| ES | GGGT | | | | | |
| PDBID:2B29 | | | | | | |
| SEG | DTNI | GN | DGL | EQLSSN | LKDG | SAEAVG |
| PDBID:2FKE | | | | | | |
| DGRT | EDG | GK | SVG | ATG | PGI | PPH |
| PDBID:2FKL | | | | | | |
| DKC | RMDV | ID | PDKC | RMDV | ID | |
| PDBID:2FMB | | | | | | |
| LEKR | ND | DTGADTS | LKYR | VG | KG | DI |
| PDBID:2I9V | | | | | | |
| AFGS | DDG | DGDG | DPKQVIG | DSPEF | DQQM | ALSG |
| PDBID:2IGP | | | | | | |
| PLND | QAP | CPSY | DTK | VGAPH | | |
| PDBID:2PK7 | | | | | | |
| CPICK | SADK | DG | CPICK | SADK | DG | |
| PDBID:2PWS | | | | | | |
| GCY | WG | NG | LY | | | |
| PDBID:2QAS | | | | | | |
| DLMQ | APGG | PEPH | TKAAGV | YPD | QHQ | GET |
| GG | FSA | | | | | |
| PDBID:2QVT | | | | | | |
| SPTA | SGL | NEQM | QAEE | GPKLNL | SNMDQH | SGAT |
| NDIPNF | | | | | | |
| PDBID:2VKS | | | | | | |

| | | | | | | |
|---|---|---|---|---|---|---|
| GLFC | ED | SVA | SFI | IVRV | | |
| PDBID:3OZZ | | | | | | |
| AK | HY | LSE-HPGG | AG | | | |
| PDBID:3UNN | | | | | | |
| AH | MPDCS | WD | CGSLNG | RPP | RDQ | AD |
| PDBID:3V19 | | | | | | |
| HSI | | | | | | |
| PDBID:3VYA | | | | | | |
| ED | DGN | GGM | RDE | RKV | RN | IARFKWA |
| PDBID:4AHI | | | | | | |
| DAKPQGR | GLTSP-CID | ENKNG | REN | GGS | WPPC | NG |
| PDBID:4AQI | | | | | | |
| GRDG | DKNE | | | | | |
| PDBID:4AQJ | | | | | | |
| RRDG | NYLA | DKNE | | | | |
| PDBID:4EX0 | | | | | | |
| TSI | | | | | | |
| PDBID:4LYO | | | | | | |
| LDN | NF | TQA | NTDG | GILQ | SRWW | DGRTPGS |
| NLCN | SSD | GNGMNAW | KGTD | IRGC | | |
| PDBID:4UNG | | | | | | |
| TSI | TSI | | | | | |
| PDBID:5AFG | | | | | | |
| DAAQQH | CSND | VKE | | | | |
| PDBID:5B1G | | | | | | |
| LDN | NF | TQA | NTDG | GILQ | SRW | DGRTPGS |
| LCN | SSD | NGMNAW | KGT | RGC | | |
| PDBID:5BMH | | | | | | |
| GKTL | | | | | | |
| PDBID:5C6X | | | | | | |

| | | | | | | |
|---|---|---|---|---|---|---|
| AAA | AV | EA | DV | DGPG | TD | SGA |
| PDBID:3A0S | | | | | | |
| KDG | VLG | LPD | GE | NAKTQ | | |
| PDBID:3BIA | | | | | | |
| LG | PNSKCNA | DGTR | STK | SN | GWFN | |
| PDBID:3C4S | | | | | | |
| FPG | NVDDTYYR | DG | GN | FPG | NVDDTYYR | DG |
| GN | | | | | | |
| PDBID:3C5K | | | | | | |
| PWCPH | PAAG | DVTQ | CGDCG | IQE | LSCY | GRYING |
| YIDL | YYCQ | GEDM | | | | |
| PDBID:3C7I | | | | | | |
| HPW | KI | HDG | ESAPG | GN | DGAG | LWV |
| RST | SRNQ | | | | | |
| PDBID:3DJN | | | | | | |
| NHK | PSE | PY | GEDG | | | |
| PDBID:3FFY | | | | | | |
| NAT | PNEK | PQKKGR | VDE | GPER | KI | |
| PDBID:3GKY | | | | | | |
| HSI | HSI | GERG | | | | |
| PDBID:3HQB | | | | | | |
| YKH | NNDE | NNDE | | | | |
| PDBID:3IN2 | | | | | | |
| AECS | DQM | NTN | DKSCKQ | PKNVMG | TAAD | GSG |
| FPGHSALL | | | | | | |
| PDBID:3LR0 | | | | | | |
| DPD | EKTD | GDDT | ND | DD | | |
| PDBID:3LVE | | | | | | |
| PD | SLG | SQS | SSNS | KPGQ | WAS | ESGVPDR |
| GT | SS | QAEDV | YYSTPY | | | |

| | | | | | | |
|---|---|---|---|---|---|---|
| PD | PG | AEPP | GAGE | GPGG | | |
| PDBID:5CUL | | | | | | |
| SS | PTH | RRGETPLP | NVD | | | |
| PDBID:5D53 | | | | | | |
| TSI | FVNQ | | | | | |
| PDBID:5FD1 | | | | | | |
| IKCK | CPVD | PN | HPDEC | CPAQ | DGVKGK | |
| PDBID:5KAZ | | | | | | |
| LPY PS | HGRL | VDG | SESIPG | KN | EKHGY | AEGS |
| PDBID:5NGN | | | | | | |
| DSCSEYC | CCP | DSCSEYC | DGQ | CCP | | |
| PDBID:5OC4 | | | | | | |
| PAQI | AE | | | | | |
| PDBID:5PAZ | | | | | | |
| AE | PA | NPG | VDK | IKDM | PEGA | KINE |
| CTAH | GDSP | SA | | | | |
| PDBID:5TAB | | | | | | |
| GPLGSEV | RCICE | NDF | CEECQ | CYVC | | |
| PDBID:5UET | | | | | | |
| DVEALGL | IKHP | PPDH | | | | |
| PDBID:5XUK | | | | | | |
| KRFK | KDK | GKELS | SPKN | AG | | |
| PDBID:5ZND | | | | | | |
| RDDVA | PDCDDW | DPHLCD | | | | |
| PDBID:6CEE | | | | | | |
| CPH | PAAG | DVTQ | CGDCG | IQE | LSCY | GRYING |
| YIDL | YYCQ | | | | | |
| PDBID:6E | | | | | | |

| | | | | | | |
|---|---|---|---|---|---|---|
| PDBID:3MF8 | | | | | | |
| QDRLT | PAGN | GG | VEYG | | | |
| PDBID:3MYA | | | | | | |
| PRGVDPSR | LVNT | PRGV | PS | | | |
| PDBID:3Q7Y | | | | | | |
| TDTG | NPDG | DRSDPGI | DGNG | TDTG | NPDG | DRSDPGI |
| DGNG | TDTG | NPDG | DRSDPGI | | | |
| PDBID:3RHE | | | | | | |
| KN DPDE | PIES | PT | VKTG | IEPKA | SNE | QDF |
| PDBID:3STM | | | | | | |
| KGKDI | GK | GS | TMTG | EGDN | KN | GD |
| GD | | | | | | |
| PDBID:3SUL | | | | | | |
| NADQ | VACS | GPNG | SGTG | AGNG | NGQ | |
| PDBID:3T1X | | | | | | |
| DRKG | ALWA | PPP | LLG | GERM | GEHA | DETA |
| PDBID:3WW5 | | | | | | |
| LDN | NF | TQA | ETDG | GILQ | SRW | DGRTPGS |
| LCN | SSD | DGNGMNAW | KGTD | RGC | | |
| PDBID:3ZEK | | | | | | |
| LDN | NF | TQA | NTDG | GILQ | SRWW | DGRTPGS |
| NLCN | SSD | DGNGMNAW | KGTD | RGC | | |
| PDBID:4AHG | | | | | | |
| TSPC | ENKN | REN | GG | WPPC | NG | |
| PDBID:4ET9 | | | | | | |
| LDN | NF | TQA | NTDG | GILQ | SRW | DGRTPGS |
| LCN | SSD | DGNGMNAW | KGTD | IRGC | | |
| PDBID:4GF | | | | | | |

| | | | | | | |
|---|---|---|---|---|---|---|
| KB | | | | | | |
| CAN-CEGEG | CSQCKGG | HFNG | KAG | CWLCRGK | CGDCNGA | |
| PDBID:6I3S | | | | | | |
| EKQQH | CSND | VKE | | | | |
| PDBID:6QQJ | | | | | | |
| DPRLPDN | ILG | GPET | TKSG | DQKG | | |
| PDBID:6RNT | | | | | | |
| CD | GS | GSNS | NYEGFDF | LSSG | ENN | HTGASGNN |
| PDBID:1B2O | | | | | | |
| TVC | NPGT | PDDW | CPLCA | TVC | NPGT | PDDW |
| CPLCA | | | | | | |
| PDBID:1BKF | | | | | | |
| DGRT | KRG | EDG | NK | GK | SVG | ATG |
| PGI | PPH | | | | | |
| PDBID:1BPQ | | | | | | |
| IPSS | NN | GCY | NPYTN | NN | SSEN | NLDKKN |
| PDBID:1C0C | | | | | | |
| KET | DSST | NLT-KDRC | CKNGQTN | KYPN | GNPY | |
| PDBID:1C9H | | | | | | |
| DGRT | KKG | QNG | SRDRN | GK | SLG | ATG |
| PGV | PPN | | | | | |
| PDBID:1DMM | | | | | | |
| GD | DPFGQ | SHNG | NG | DEHG | | |
| PDBID:1DMQ | | | | | | |
| DPFGQ | SHNG | NG | DEHG | | | |
| PDBID:1DYZ | | | | | | |
| AQC | NDAM | NVK | DKSCK | AK-VAMG | GGG | FPGH-WAMM |
| PDBID:1DZ0 | | | | | | |
| AQC | NDAM | NVK | DKSCK | AK-VAMG | GGG | FPGH-WAMM |
| PDBID:1E6M | | | | | | |
| SDKEL | DDF | WNMPNMD | DGAMSAL | KPF | | |

| | | | | | | |
|---|---|---|---|---|---|---|
| Y | | | | | | |
| SS | GCY | GWG | VNGA | LYPD-FLCK | | |
| PDBID:4HMB | | | | | | |
| GCY | WG | NG | LY | | | |
| PDBID:4HP9 | | | | | | |
| FQSR | ARVENC | MQ | CTC | GPRT | RKDG | NEDG |
| PDBID:4HRS | | | | | | |
| GR | VVGE | DDDG | DG | PEDQ | VEV | RLIK |
| PDBID:4IAS | | | | | | |
| LDN | NF | TQA | NTDG | GILQ | SRW | DGRTPGS |
| LCN | SSD | DGN | KGTD | RGC | | |
| PDBID:4KUO | | | | | | |
| DPSQPDN | VLG | GPDT | RKDG | DPEG | | |
| PDBID:4LFS | | | | | | |
| SCI | RKTCG | | | | | |
| PDBID:4ML2 | | | | | | |
| TLPL | KG | PD | TDK | | | |
| PDBID:4P9V | | | | | | |
| HPW | KI | HDG | ESAPG | GN | DGAG | LWV |
| SRNQ | | | | | | |
| PDBID:4PTA | | | | | | |
| NW | DTEG | KRPN | | | | |
| PDBID:4XC4 | | | | | | |
| TSI | | | | | | |
| PDBID:5AEF | | | | | | |
| GVVI | GVVI | | | | | |
| PDBID:5CB9 | | | | | | |
| PD | PA | PG | AEPP | GAGE | NDTACCYA | GPGG |
| PDBID:5J6X | | | | | | |
| DN | KQ | DN | | | | |
| PDBID:5K2L | | | | | | |

| | | | | | | |
|---|---|---|---|---|---|---|
| PDBID:1E97 | | | | | | |
| DPFGQ | SHNG | NG | DEHG | | | |
| PDBID:1EA2 | | | | | | |
| DPFGQ | SHNG | NG | DEHG | | | |
| PDBID:1ED1 | | | | | | |
| LRPGG | VPTG | TGTA | | | | |
| PDBID:1FAV | | | | | | |
| EL | | | | | | |
| PDBID:1G2S | | | | | | |
| SDISKT | SNSCSQR | KRG | HPRK | TPK | | |
| PDBID:1HKF | | | | | | |
| VAGQ | TGSL | AL | KPR | TSR | TDL | RPSDN |
| PDBID:1I0C | | | | | | |
| NSS | MKD | RQ | NASG | NSS | MKD | RQ |
| NASG | NNI | | | | | |
| PDBID:1IOT | | | | | | |
| LDN | NF | TQA | NTDG | GILQ | SRWW | DGRTPGS |
| NLCN | SSD | DGN | KGTD | RGC | | |
| PDBID:1J4H | | | | | | |
| DGRT | KRG | EDG | GKQEVI | SVG | ATG | PGI |
| PPH | | | | | | |
| PDBID:1JC7 | | | | | | |
| AN | PVHH | GGN | GDPLICDN | STG | WPAHRNE | |
| PDBID:1JHC | | | | | | |
| AA-GEPLLA | DPSLFKPN | MA | MDG | QDVRNG | DD | GN |
| NSEF | LRQQ | DWLV | | | | |
| PDBID:1JON | | | | | | |
| DR | LS | IN | SP | GIVK | NKDT | |
| PDBID:1KC2 | | | | | | |

| | | | | | | |
|---|---|---|---|---|---|---|
| QPGD | | | | | | |
| PDBID:5O3A | | | | | | |
| IKHP | PPDH | | | | | |
| PDBID:5UER | | | | | | |
| DVEALG | IKHP | PPDH | | | | |
| PDBID:5VEA | | | | | | |
| LWKRP | GG | DTGADD | IG | CG | PTPV | |
| PDBID:5WPB | | | | | | |
| CPH | PAAG | DVTQ | CGDCG | IQE | LSCY | GRYING |
| YIDL | YYCQ | | | | | |
| PDBID:5WRB | | | | | | |
| LDN | NF | TQA | NTDG | GILQ | SRW | DGRTPGS |
| LCN | SSD | DGNGMNAW | KGT | RGC | | |
| PDBID:6FH7 | | | | | | |
| SMSV | HED-AWPFL | NLKLVPG | IKKP | EDDS | | |
| PDBID:6MHN | | | | | | |
| AFGS | GDG | DPKQVIG | DSPEF | DYQM | ALSGD | |
| PDBID:8PAZ | | | | | | |
| AE | PA | NPG | VDK | IKDM | PEGA | KINE |
| CTPH | GDSP | | | | | |
| PDBID:134L | | | | | | |
| MDG | GY | TRA | AGDR | GIFQ | SRYW | DGKTPGA |
| NACH | QDN | DPQ | QNR | VQGC | | |
| PDBID:1A6F | | | | | | |
| NRQ | QPEN | SKKIG | ER | KEK | SQL | |
| PDBID:1BEL | | | | | | |
| DSSTS | NLT-KDRC | CKNG | TGSS | YP | GNPY | |
| PDBID:1BGI | | | | | | |
| LDN | NF | TQA | NTDG | GILQ | SRWW | DGRTPGS |

| | | | | | | | | | | | | | |
|---|---|---|---|---|---|---|---|---|---|---|---|---|---|
| EEW | KI | NPEN-PRG | SETTKGA | LNV | LDSG | SR | NLCN | SSD | NGMNAW | KGT | RGC | | |
| ADGLCHR | | | | | | | PDBID:1BO0 | | | | | | |
| PDBID:1KP4 | | | | | | | PVGI | TSSHCPRE | KLD | KKT | TPK | | |
| DLCTQA | NPFGFP | | | | | | PDBID:1BYL | | | | | | |
| PDBID:1KXW | | | | | | | RD | DD | DD | DQVV | RG | TNFRDA | PW |
| LDN | NF | TQA | NTDG | GILQ | SRWW | DGRTPGS | DPAG | | | | | | |
| NLCN | SSD | DGN | KGTD | RGC | | | PDBID:1EIF | | | | | | |
| PDBID:1LSL | | | | | | | KVG | DG | IFE | TSS | GD | LQTY | PEGI |
| SVTC | QMNG | EA | VTCG | FGG | DV | NK | EPG | VG | | | | | |
| PDBID:1LYO | | | | | | | PDBID:1FKV | | | | | | |
| LDN | NF | TQA | NTDG | GILQ | SRWW | DGRTPGS | KD | GY | TQA | NNDS | GLFQ | NKIW | DDQNPHS |
| NLCN | SSD | GN | KGTD | IRGC | | | NICN | | | | | | |
| PDBID:1O4H | | | | | | | PDBID:1GP3 | | | | | | |
| EEW | KI | NAENPRG | SETTKG | NAK | LDSG | SR | NPSTG | SEK | CG | NENCPPG | GQTR | KR | DQ |
| ADGLCHR | | | | | | | CPSKSGL | KQTC | | | | | |
| PDBID:1OAP | | | | | | | PDBID:1GS3 | | | | | | |
| QQNN | DLDK | NPSY | KEK-PAVL | | | | GD | NPFGQ | SHNG | NG | DEHG | | |
| PDBID:1QTO | | | | | | | PDBID:1GSW | | | | | | |
| VD | DRD | GD | TD | RAV | DY-ADTSG | PA | AFGSED | DDG | GDG | IG | DSPE | DYQM | ALSGD |
| DPAG | | | | | | | PDBID:1HFX | | | | | | |
| PDBID:1RMD | | | | | | | NDLAG | GY | TQA | SD | GLFQ | DKDF | NICD |
| CQICE | LAD | TSCK | CPSCR | AQDC | | | PDBID:1I9E | | | | | | |
| PDBID:1SV9 | | | | | | | EG | YPRQ | VNG | KSNS | AS | HWSDS | FA |
| SS | GCY | GWG | NG | LY | | | PDBID:1IGU | | | | | | |
| PDBID:1U3Y | | | | | | | DPD | EDDG | DE | LN | AEG | EDDG | DPD |
| DRT | VTG | KD | RQF | YKDVNI | RKSDL | | LKK | DE | LN | PPAEG | | | |
| PDBID:1UIE | | | | | | | PDBID:1IR8 | | | | | | |
| LDN | NF | TQA | NTDG | GILQ | SRWW | DGRTPGS | LDN | NF | TQA | NTDG | GILQ | SRWW | DGRTPGS |
| NLCN | SSD | DGN | KGT | RGC | | | NLCN | SSD | DGN | KGTD | RGC | | |
| PDBID:1UIF | | | | | | | PDBID:1JOI | | | | | | |
| LDN | NF | TQA | NTDG | GILQ | SRWW | DGRTPGS | AEC | TDQM | DKSCK | PKNVMG | GAG | AAGT | FPGHISMM |

| | | | | | | |
|---|---|---|---|---|---|---|
| NLCN | SSD | GN | KGTD | RGC | | |
| PDBID:1W2L | | | | | | |
| SIDG | RLVGP | FKGLYG | EDG | QPGAKV | QGYPNV | |
| PDBID:1WJX | | | | | | |
| FTGS | DG | NL | PVDPRR | LGKVE | KG | NERG |
| PDBID:1Y49 | | | | | | |
| FCNAFTG | | | | | | |
| PDBID:1Y9T | | | | | | |
| FSIPNDL | TTNK | SKC | AG | VDGC | NKIN | DG |
| PDBID:1Z3P | | | | | | |
| NLTKDR | CKNG | TGSS | YP | GNPY | | |
| PDBID:2A7B | | | | | | |
| NG | SNTNPS | PKTF | PAFNT | NPQK | KG | NNF |
| PDBID:2AZB | | | | | | |
| LWKRP | GG | DTGA | CG | TPA | | |
| PDBID:2B1Y | | | | | | |
| RAGT | KSPI | AEGH | | | | |
| PDBID:2BFH | | | | | | |
| KNGG | HPDG | EKS-DPHI | RG | VCAN | KEDG | KCVTDEC |
| ESNN | RKYTSW | KRTG | GPGQKAI | | | |
| PDBID:2EYW | | | | | | |
| GAM | DEED | KKGD | KPE | DSEG | VPYV | PASA |
| PDBID:2GV2 | | | | | | |
| DEKQQH | CSND | VKE | | | | |
| PDBID:2H2B | | | | | | |
| GSHM | PGF | FG | NG | MDNV | SGK | WRRT |
| PDBID:2H36 | | | | | | |
| MEKV | LGNY | NG | DDD | EDN | | |
| PDBID:2IIY | | | | | | |
| AGDK | ATWC | YSN | VDD | CMP | KG | GAN |

| | | | | | | |
|---|---|---|---|---|---|---|
| PDBID:1K58 | | | | | | |
| DAKPQGR | LTSPCKD | ENKN | REN | WPPC | NG | HQSIFR |
| PDBID:1KJT | | | | | | |
| YPD | APKARI | LDKK | SDL | RAED | NNV | PTSA |
| EEDF | | | | | | |
| PDBID:1KXY | | | | | | |
| LNN | NF | TQA | NTDG | GILQ | SRWW | DGRTPGS |
| NLCN | SSD | DGN | KGTD | RGC | | |
| PDBID:1LAC | | | | | | |
| KLPD | EGIH | KPG | NEDD | NDKA | SPVK | LVP |
| VGQT | | | | | | |
| PDBID:1MKU | | | | | | |
| IPSS | NN | GCY | NPYTN | NN | SSEN | NLDKKN |
| PDBID:1NXT | | | | | | |
| PD | MLPD | AD | KPF | | | |
| PDBID:1O43 | | | | | | |
| EEW | KI | NAENPRG | SETTKG | NAK | LDSG | SR |
| NS | ADGLCHR | | | | | |
| PDBID:1O44 | | | | | | |
| EEW | KI | NAENPRG | SETTKGA | NAK | LDSG | SR |
| ADGLCHR | | | | | | |
| PDBID:1O4A | | | | | | |
| EEW | KI | NAENPRG | SETTKGA | NAK | LDSG | SR |
| ADGLCHR | | | | | | |
| PDBID:1O4I | | | | | | |
| EEW | KI | NAENPRG | SETTKGA | NAK | LDSG | SR |
| ADGLCHR | | | | | | |
| PDBID:1Q4V | | | | | | |
| GV | SLYQL | GERG | GERG | | | |
| PDBID:1QKF | | | | | | |

| | | | | | | |
|---|---|---|---|---|---|---|
| PDBID:2OXK | | | | | | |
| EK | | | | | | |
| PDBID:2P0F | | | | | | |
| GG | GN | REPPPTA | GWGPAGS | LRGA | GRHLS | RR |
| TIPG | DH | | | | | |
| PDBID:2PKT | | | | | | |
| TPGS | CIG | DG | TSNM | CTD | | |
| PDBID:2QHE | | | | | | |
| NG | TYY | | | | | |
| PDBID:2R2Y | | | | | | |
| YL | GT | TDDS | RTSG | FPDD | PQCPSG | KAGS |
| EPKT | | | | | | |
| PDBID:2R34 | | | | | | |
| TSI | TPK | TSI | GERG | | | |
| PDBID:2R36 | | | | | | |
| TSI | GERG | PKT | TSI | LENY | GERG | |
| PDBID:2SAK | | | | | | |
| DSKG | KPG | TAYKE | DPSA | KNKK | TEKG | LSE-HIKNP |
| PDBID:2WOR | | | | | | |
| RRDD | NYLA | DKNE | | | | |
| PDBID:2WOS | | | | | | |
| RRDD | NYLA | DKNE | | | | |
| PDBID:2X9C | | | | | | |
| KPSD | KPSD | | | | | |
| PDBID:2XKH | | | | | | |
| VN | FGFKGV | | | | | |
| PDBID:3AZ5 | | | | | | |
| LDN | RG | NF | TQA | NTDG | GILQ | SRWW |
| DGRTPGS | NLCN | SSD | NGMNAW | KGT | RGC | |
| PDBID:3B84 | | | | | | |
| SRR | VG | NGKQ | | | | |
| PDBID:1R75 | | | | | | |
| FKNG | MFSNI | TEAR | SKE | YDS | LGDT | IG |
| RPL | AEDERG | | | | | |
| PDBID:1RAQ | | | | | | |
| LQCH | DQGG | NKVGP | LHGIFG | SGQAEGY | IPGT | |
| PDBID:1RBI | | | | | | |
| NLTKDR | CKNG | TGSS | YP | GNPY | | |
| PDBID:1RBW | | | | | | |
| DSST | NLT-KDR | CKNGQTN | TGSS | YP | GNPY | |
| PDBID:1RNN | | | | | | |
| DSSTS | NLT-KDR | CKNG | TGSS | YP | GNPY | |
| PDBID:1RNU | | | | | | |
| NLTKDR | CKNG | TGSS | YP | GNPY | | |
| PDBID:1RNW | | | | | | |
| DSST | NLT-KDR | CKNG | TGSS | YP | GNPY | |
| PDBID:1RWJ | | | | | | |
| KGMT | PK | MKGV | GMY | CHT-KLF | KAGAKR | |
| PDBID:1S7I | | | | | | |
| LAAV | QGGR | TKE | | | | |
| PDBID:1SFG | | | | | | |
| LA | LDN | RG | NTDG | GILQ | SRWW | DGRTPGS |
| NLCN | SSDITA | NRCKGT | WIRGC | | | |
| PDBID:1TDY | | | | | | |
| MDG | GY | TRA | AGDR | GIFQ | SRWW | DGKTPGA |
| NACH | QDN | DPQ | QNR | VQGC | | |
| PDBID:1TEN | | | | | | |
| TDT | KDVPGD | EDE | KPDT | GD | | |
| PDBID:1VAT | | | | | | |
| LDN | NF | TQA | NTDG | GILQ | SRW | DGRTPGS |

| | | | | | | |
|---|---|---|---|---|---|---|
| GQYC | GG | GDG | LALTSG | | | |
| PDBID:3BYR | | | | | | |
| MDEG | RGRA | GP | RGDT | FPG | | |
| PDBID:3BZS | | | | | | |
| ST | PTH | KLGETPLP | HKG | | | |
| PDBID:3C12 | | | | | | |
| DATG | DANG | DANG | TAG | SD | TGL | LANV |
| PDBID:3CTG | | | | | | |
| KE | KTYC | ELN | TVP | NG | PVF | |
| PDBID:3CYI | | | | | | |
| GAQG | | | | | | |
| PDBID:3HNU | | | | | | |
| NADG | DG | GG | GAAE | DCNG | LNRI | NG |
| PDBID:3KMJ | | | | | | |
| NDR | SPLG | RKS | EKG | TALE-DY | KN | LGF-GAIF |
| KDP | TAGLK | IKP | AIG | GDD | RLTQ | |
| PDBID:3KQ6 | | | | | | |
| HSI | HSI | | | | | |
| PDBID:3LZ2 | | | | | | |
| LDN | NF | THA | NTDGS | GILQ | SRWW | TPG |
| NLCN | SSDI | GGNGMNAW | KGT | IRGC | | |
| PDBID:3OSE | | | | | | |
| TWSM | RF | DARQD | | | | |
| PDBID:3RTO | | | | | | |
| TSI | | | | | | |
| PDBID:3S8S | | | | | | |
| GQIP | RLNDNV | HPRTR | TS | HL | MG | DIKG |
| TPQTV | | | | | | |
| PDBID:3WIT | | | | | | |
| NG | EE | PD | GES | KD | GGS | |
| PDBID:3WRP | | | | | | |

| | | | | | | |
|---|---|---|---|---|---|---|
| LCN | SSD | DGN | KGTD | RGC | | |
| PDBID:1YEA | | | | | | |
| RC | EEGG | NKVGP | LHG | SGQVKGY | IPGT | |
| PDBID:1YMV | | | | | | |
| MPNMD | DGAMSAL | EAK | PF | | | |
| PDBID:1YVS | | | | | | |
| PDNY | VAPGK | NREGKLP | SG | NY | SDW | TDHYQT |
| PDBID:1Z3M | | | | | | |
| NLTKDR | CKNG | TGSS | YP | GNPY | | |
| PDBID:2BWK | | | | | | |
| DAKPKGR | TSPC | GAN | REN | GG | RPPC | NG |
| PDBID:2BWL | | | | | | |
| DAKPKGR | TSPC | GAN | REN | RPPC | NG | ESF |
| PDBID:2D4L | | | | | | |
| ATPGS | PPN | DNDY | VNN | SQG | | |
| PDBID:2DYQ | | | | | | |
| AR | SDSL | PL | GRDPH | LGRQS | QPH | |
| PDBID:2F3L | | | | | | |
| DV | LIGE | FSGK | LTYA | NA | LTDS | FSEA |
| LRGA | GS | LIGA | LHGA | LTNG | FKGA | LTNA |
| LTEA | FDDA | ITGA | FSLA | NPKTG | | |
| PDBID:2F4G | | | | | | |
| LDN | NF | TQA | NTDG | GILQ | SRWW | DGRTPGS |
| NLCN | SSD | NGMNAW | KGTD | RGC | | |
| PDBID:2FB0 | | | | | | |
| NET | EEG | SSTRRD | | | | |
| PDBID:2FQL | | | | | | |
| SHG | PAF | PPNKQ | PLS | LNGE | LRNG | |
| PDBID:2M6D | | | | | | |
| CVYP | | | | | | |
| PDBID:2M6E | | | | | | |

| | | | | | | |
|---|---|---|---|---|---|---|
| SAA | LKS | | | | | |
| PDBID:3WX4 | | | | | | |
| AG | APGK | | | | | |
| PDBID:4DP1 | | | | | | |
| ADDGSLA | PS | PAG | SEED | AKG | CSPH | GM |
| PDBID:4F1A | | | | | | |
| TSI | | | | | | |
| PDBID:4IP1 | | | | | | |
| KGK | ADWC | FSD | ADT | VTAN | GLP | DGQG |
| PDBID:4OV1 | | | | | | |
| DQDKC | APEL | DDYG | KGDG | PAD | CPEN | |
| PDBID:4OZL | | | | | | |
| ADTP | EKGD | VRTG | | | | |
| PDBID:4PAZ | | | | | | |
| AE | PA | NPG | VDK | IKDM | PEGA | KINE |
| CTAH | GDSP | | | | | |
| PDBID:4TS8 | | | | | | |
| QDPES | DPQLLG | VKNP | GQYQE | RKTS | | |
| PDBID:4XXL | | | | | | |
| GVDK | KDGP | RGK | FPDG | INGK | | |
| PDBID:5B52 | | | | | | |
| RLA | | | | | | |
| PDBID:5ER4 | | | | | | |
| EGDKH | SHFL | DSDG | | | | |
| PDBID:5GSP | | | | | | |
| CD | GS | GSNS | NYEG | LSSG | ENN | HTGASGNN |
| PDBID:5LAZ | | | | | | |
| EKQQH | CSND | VKE | | | | |
| PDBID:5UES | | | | | | |
| DVEAL-GLH | IKHP | PPDH | | | | |
| PDBID:5YI1 | | | | | | |
| ATNDERV | | | | | | |
| PDBID:6CED | | | | | | |

| | | | | | | |
|---|---|---|---|---|---|---|
| GC | CV | VY | YP | PW | WC | |
| PDBID:2M6G | | | | | | |
| GCPD | | | | | | |
| PDBID:2OLI | | | | | | |
| GCY | NG | LY | | | | |
| PDBID:2P6V | | | | | | |
| TPD | | | | | | |
| PDBID:2PW5 | | | | | | |
| GD | KG | LL | ETKVEKY | AKK | DKYG | DG |
| KG | | | | | | |
| PDBID:2PYK | | | | | | |
| GD | KG | LL | ETKVEKY | AKK | DKYG | DG |
| KG | | | | | | |
| PDBID:2PZW | | | | | | |
| GD | KG | LL | ETKVEKY | AKK | DKYG | DG |
| KG | | | | | | |
| PDBID:2RKN | | | | | | |
| CG | KENP | YKNS | PT | TC | | |
| PDBID:2RNS | | | | | | |
| NLTKDR | CKNG | TGSS | YP | GNPY | | |
| PDBID:2VJW | | | | | | |
| AV | EA | DV | DGPG | TD | SGA | |
| PDBID:2VP7 | | | | | | |
| SSSD | GICT | NDDQ | EASC | EAS | CDTCM | TE |
| GQVETIVS | | | | | | |
| PDBID:2XDY | | | | | | |
| GSKANEG | HQ | PRK | TFGV | STKG | | |
| PDBID:2XFE | | | | | | |
| VNG | GG | TG | NG | TGV | NN | GG |
| HCN | | | | | | |
| PDBID:2XMU | | | | | | |
| VPTI | DAQA | LTSK | VPTI | EDAQA | LTSK | |
| PDBID:2YXY | | | | | | |

| | | | | | | |
|---|---|---|---|---|---|---|
| CPH | PAAG | DVTQ | CGDCG | IQE | LSCY | GRYING |
| YIDL | YYCQ | | | | | |
| PDBID:6FGL | | | | | | |
| HDAAWPFL | NPRLVSG | IKNP | EDDS | | | |
| PDBID:6H0K | | | | | | |
| LDN | NF | TQA | NTDG | GILQ | SRWW | DGRTPGS |
| NLCN | SSD | DGNGMNAW | KGTD | IRGC | | |
| PDBID:155C | | | | | | |
| NEG | KCKAC | GKT | NPDL | GKN | AXXXX | |
| PDBID:1ANG | | | | | | |
| QDN | DAKPQGR | GLTSPCKD | ENKN | REN | WPPC | NG |
| PDBID:1AQT | | | | | | |
| AEQ | SEG | YPGH | QHG | PG | SHG | |
| PDBID:1BXY | | | | | | |
| PIGY | RLQ | PIGY | RLQ | AHL | | |
| PDBID:1CDP | | | | | | |
| FAGVL | AADS | GLTSK | DQDK | FKADA | DSDG | |
| PDBID:1CDT | | | | | | |
| KLIPIA | PEGKN | ASKKM | SAL | TDRC | KLIPIA | PEGK |
| ASKKM | NVC | SAL | TDRC | | | |
| PDBID:1DPY | | | | | | |
| GCY | PG | NPNIK | QP | DSAD | ML | STSC |
| PDBID:1DQ7 | | | | | | |
| GDN | PY | PTPVP | DGDN | PY | PTPV | |
| PDBID:1F1W | | | | | | |
| EEW | KI | NPEN-PRG | SETTKGA | NAK | LDSG | SR |
| ADGLCHR | | | | | | |
| PDBID:1FLU | | | | | | |
| LDN | NF | TQA | NTDG | GILQ | SRWW | DARTPGS |
| NLCN | SSD | DGNGMNAW | KGTD | RGC | | |
| PDBID:1FLW | | | | | | |
| LDN | NF | TQA | NTDG | GILQ | SRWW | DGRTP |

| | | | | | | |
|---|---|---|---|---|---|---|
| HPG | EG | GV | LKG | | | |
| PDBID:2Z7J | | | | | | |
| IYFA | GHR | DPNE | YLDY | AEKS | APGQA | HGR |
| PDBID:3A0D | | | | | | |
| SPN | TG | GPS | QGDC | SG | TGGLGSGC | HNNG |
| DQSN | QQDR | | | | | |
| PDBID:3ADY | | | | | | |
| NTLTIP | PSVP | PNSQ | | | | |
| PDBID:3BF2 | | | | | | |
| LTYR | DAAGA | VTAVI | RG | | | |
| PDBID:3FZA | | | | | | |
| NP | KTWC | TVP | GG | | | |
| PDBID:3I7T | | | | | | |
| SR | GP | TD | KIR | LIAPGL | | |
| PDBID:3I7Y | | | | | | |
| DSSTS | NLT-KDR | KP | CKNG | TGSS | YP | GNPY |
| PDBID:3IJU | | | | | | |
| LDN | NF | TQA | NTDG | GILQ | SRW | DGRTPGS |
| LCN | SSD | DGN | KGTD | RGC | | |
| PDBID:3JTE | | | | | | |
| CNSI | MKMPKLS | TPHM | GHG | KPV | | |
| PDBID:3MAZ | | | | | | |
| PAC | GSDS | IDI | GQ | LEKP | RGNLR | |
| PDBID:3NGR | | | | | | |
| QSGP | KN | ATN | QPGT | DVGRNV | SVES | |
| PDBID:3R5P | | | | | | |
| TFQK | GRKTG | GG | AEKN | KK | | |
| PDBID:3RH1 | | | | | | |
| DSST | NLT-KDR | CKNG | TGSS | YP | GNAY | |
| PDBID:3RSK | | | | | | |
| DSST | NLT-KDR | CANG | TGSS | YP | GNPY | |
| PDBID:3S3 | | | | | | |

| | | | | | | | | | | | | | |
|---|---|---|---|---|---|---|---|---|---|---|---|---|---|
| | | | | | | AS | Y | | | | | | |
| NLCN | SSD | GNGM NAW | KGTD | RGC | | | GS | RTNG | LNSV | DG | GSS | TRAQ | DG |
| PDBID:1H QB | | | | | | | PDBID:3ZK 0 | | | | | | |
| SQFG | PVSEF | WDT | | | | | SPGAE | SDSM | EA | DG | DR | PAH | KSGG |
| PDBID:1H SQ | | | | | | | KL | KQG | AH | | | | |
| AQREDEL | IKS | EGG | YGGK | | | | PDBID:4ET 8 | | | | | | |
| PDBID:1I0 7 | | | | | | | LDN | NF | TQA | NTDG | GILQ | SRW | DGRTP GS |
| NSS | MKD | ASG | NNI | ARNSSE | MKD | NASG | LCN | SSD | DGNG MNA W | KGTD | RGC | | |
| NNI | | | | | | | PDBID:4FC 1 | | | | | | |
| PDBID:1IR 7 | | | | | | | PGDYA | | | | | | |
| LDN | NF | TQA | NTDG | GILQ | SRWW | DGRTP GS | PDBID:4FD X | | | | | | |
| NLCN | SSD | DGN | KGTD | RGC | | | GVEHF | AG | GVEHF | AG | | | |
| PDBID:1IS U | | | | | | | PDBID:4IP6 | | | | | | |
| NG | AQ | GASP-TA | KV | PGDNQ | APGG | CDAF | KGK | ADWC | FSD | ADT | VTAN | GLP | DGQG |
| NG | AQ | SPTA | KV | PGDNQ | APGG | CDAF | PDBID:4N0 Z | | | | | | |
| PDBID:1IZ A | | | | | | | NI | KTEC | IEKN | SVP | NK | | |
| TSI | SLYQL | FVN | TSI | | | | PDBID:4WB F | | | | | | |
| PDBID:1L CJ | | | | | | | KER | ATN | APGT | SIDENA | | | |
| EPW | FKNL | APGNT HG | SES-TAGS | QNQ | DNG | PR | PDBID:4YX 5 | | | | | | |
| SDGLCTR | | | | | | | PTN | ANGV | ND | YA | VEG | | |
| PDBID:1L PI | | | | | | | PDBID:5FG 4 | | | | | | |
| LDN | NF | TQA | NTDG | GILQ | SRWW | DGRTP GS | DTGNIFS | IKKP | AKDT | | | | |
| NLCN | SSD | DGN | KGTD | RGC | | | PDBID:5HB O | | | | | | |
| PDBID:1M 4A | | | | | | | IPE | HE | APA | ED | | | |
| KKA | RPR | | | | | | PDBID:5MX Y | | | | | | |
| PDBID:1M LI | | | | | | | GVDG | KFAD | PEM | FKEKL | | | |
| IAGH | HPSS | IAGH | HPSS | IAGH | HPSS | IAGH | PDBID:6BS Y | | | | | | |
| HPSS | IAGH | HPSS | IAGH | HPSS | IAGH | HPSS | NPE | | | | | | |
| IAGH | HPSS | IAGH | HPSS | IAGH | HPSS | | PDBID:6I5A | | | | | | |
| PDBID:1N N7 | | | | | | | QFQT | QVTV | PDSD | RYN | DT | ENQA | GTG |
| SG | YPD- | PRHE | LIPE | | | | | | | | | | |
