# Supplementary material for: Entropy-Enthalpy Compensations Fold Proteins in Precise Ways
Source: Int J Mol Sci. 2021 Sep 6;22(17):9653. doi: 10.3390/ijms22179653 (PMC8431812; doi:10.3390/ijms22179653)
Supplement: Supplementary file 1 [file ijms-22-09653-s001.zip › Supplementary2.pdf]

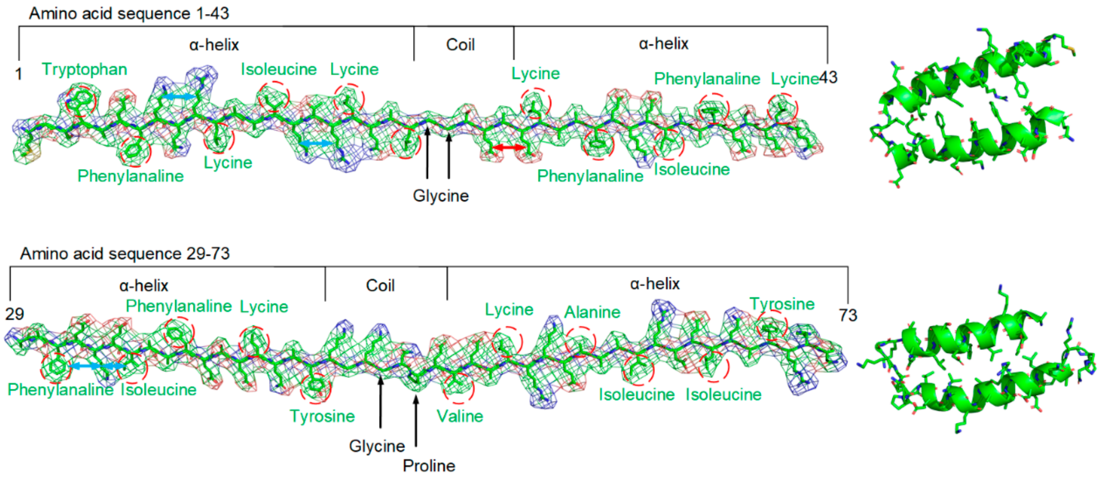

Figure 1. The folding mechanism of a protein structure (PBDID: 2A3D) based on entropy-enthalpy compensation (the hydrophobic attraction between the side-chains of adjacent residues is marked with a blue arrow, and the hydrophilic-hydrophobic and electrostatic repulsion is marked with a red arrow). The proline and glycine that led to the formation of the corner structure are marked. The hydrophobic amino acids in the sequence that cause the metastable collapse to form an alpha-helix structure are annotated by red circles.

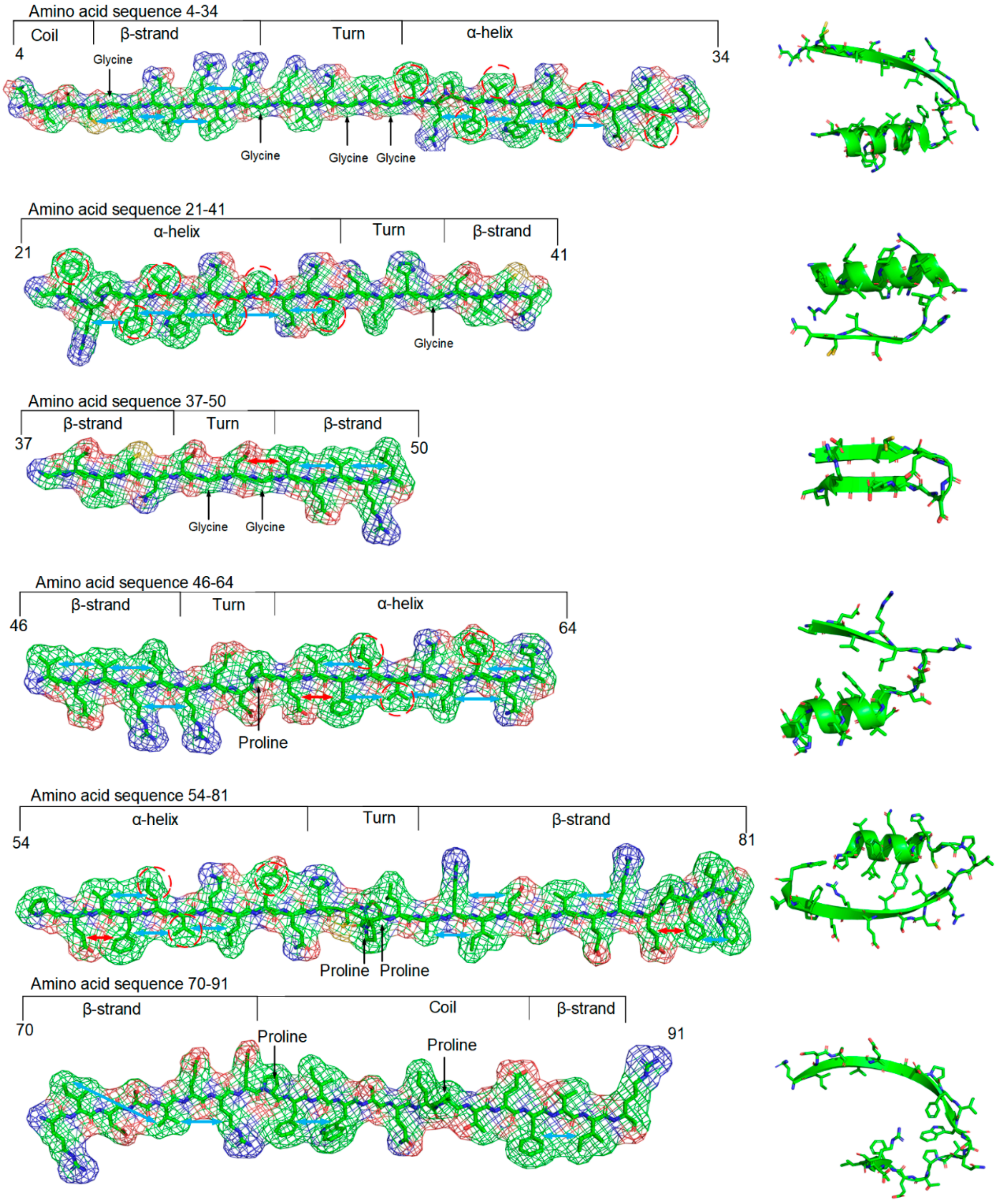

Figure 2. The folding mechanism of a protein structure (PBDID: 1GXT) based on entropy-enthalpy compensation.

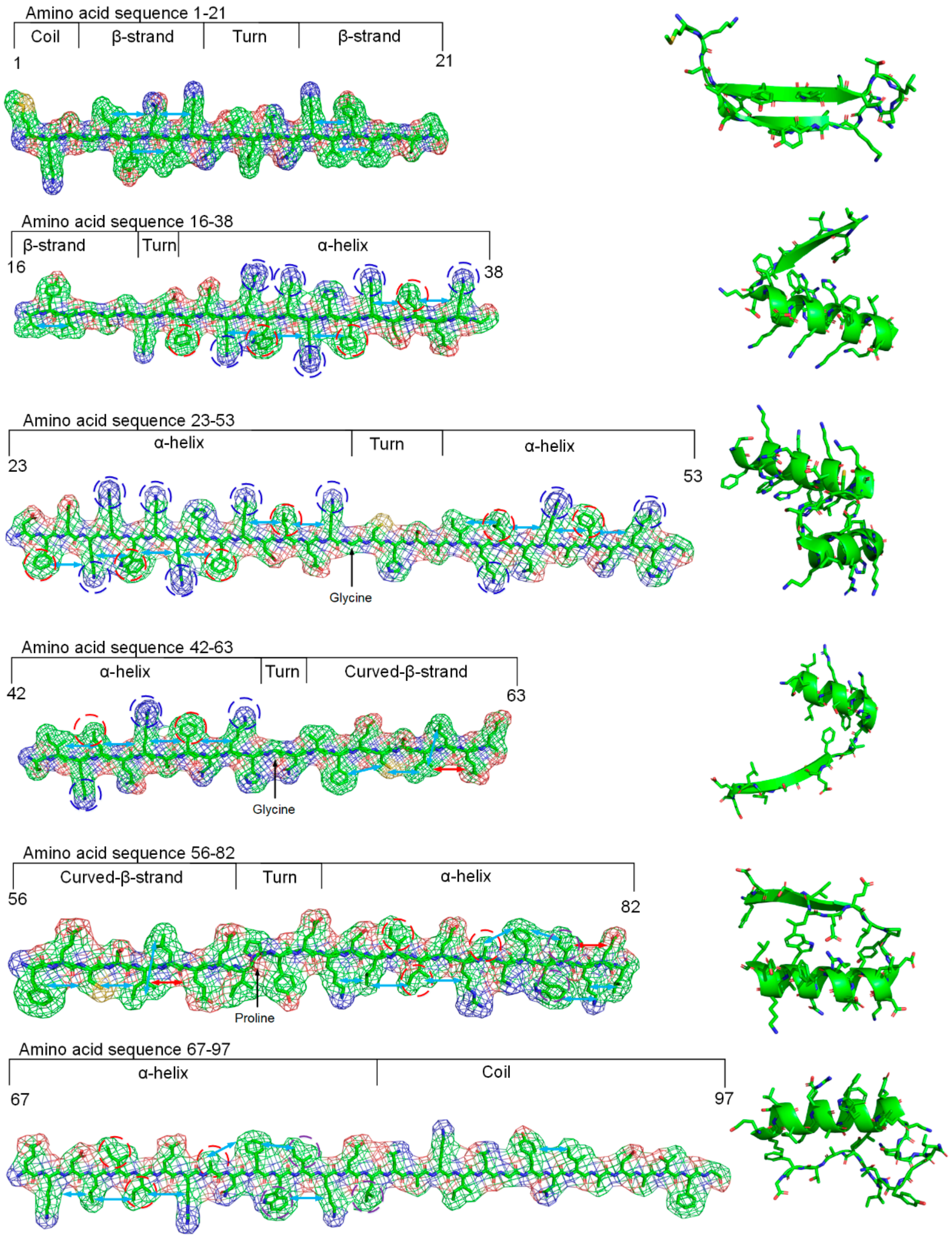

Figure 3. The folding mechanism of a protein structure (PBDID: 1MK0) based on entropy-enthalpy compensation.

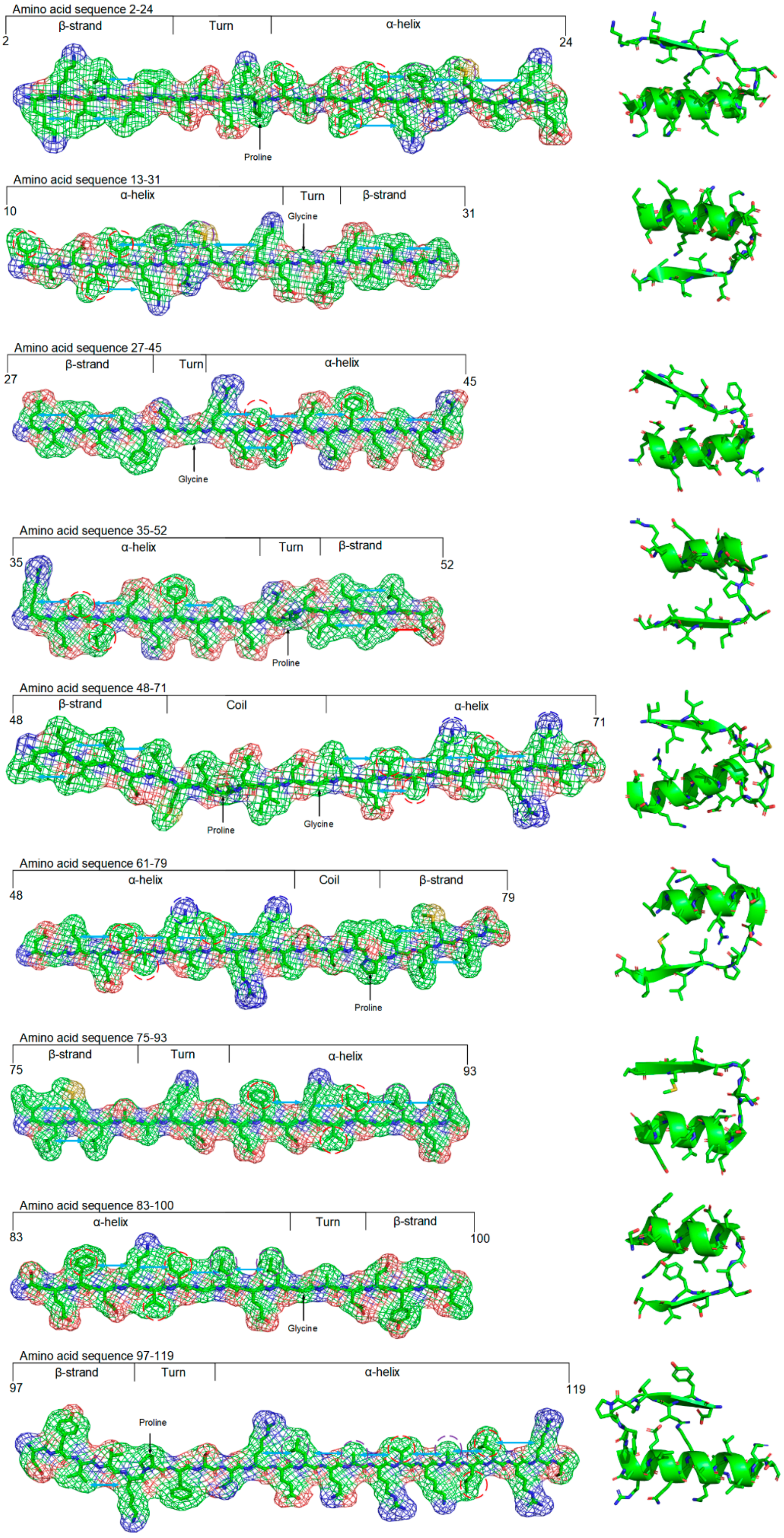

Figure 4. The folding mechanism of a protein structure (PBDID: 1NXV) based on entropy-enthalpy compensation.

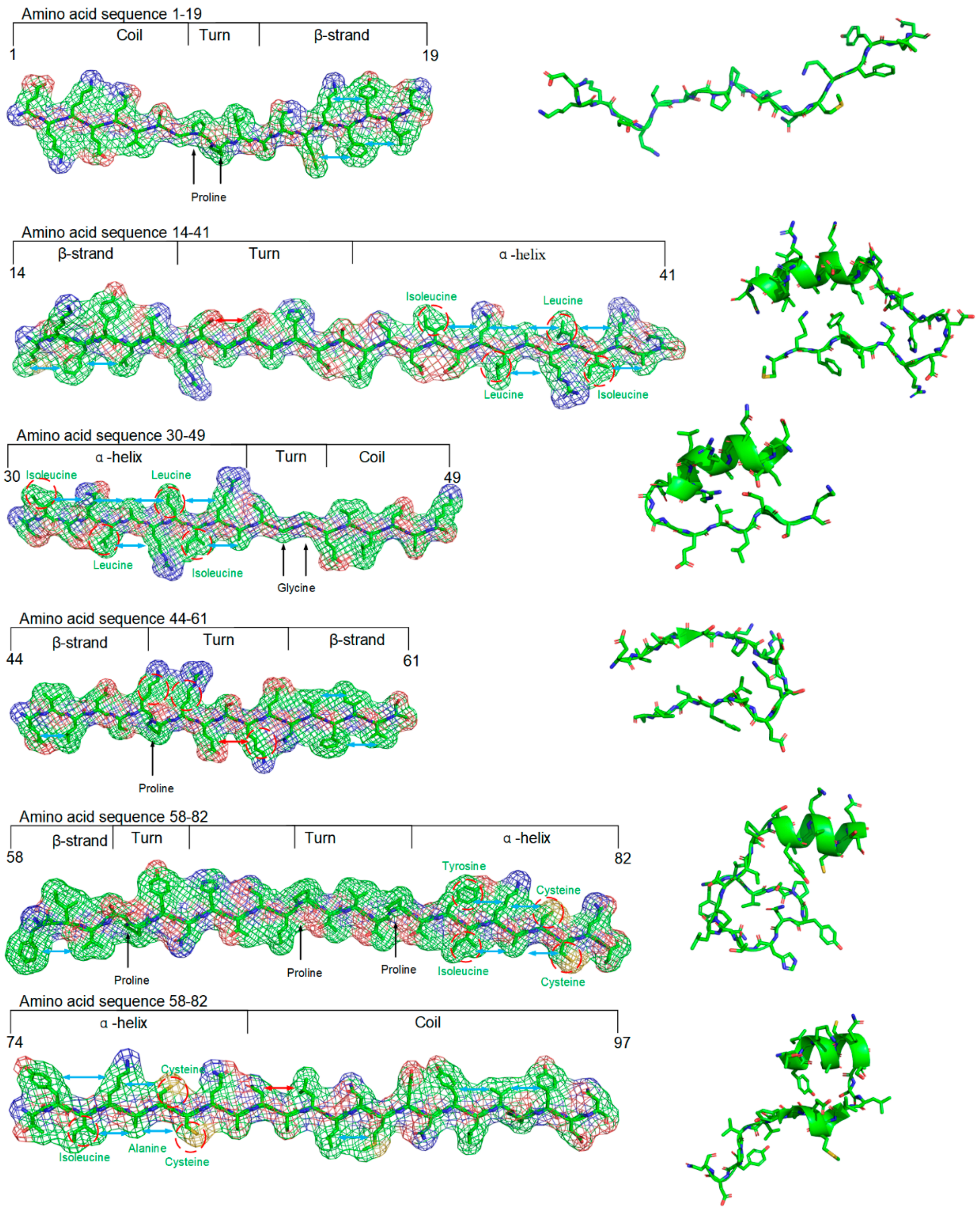

Figure 5. The folding mechanism of a protein structure (PBDID: 2L42) based on entropy-enthalpy compensation (the hydrophobic attraction between the side-chains of adjacent residues is marked with a blue arrow, and the hydrophilic-hydrophobic and electrostatic repulsion is marked with a red arrow). The proline and glycine that led to the formation of the corner structure are marked. The hydrophobic amino acids in the sequence that cause the metastable collapse to form an α-helix structure are annotated by red circles.

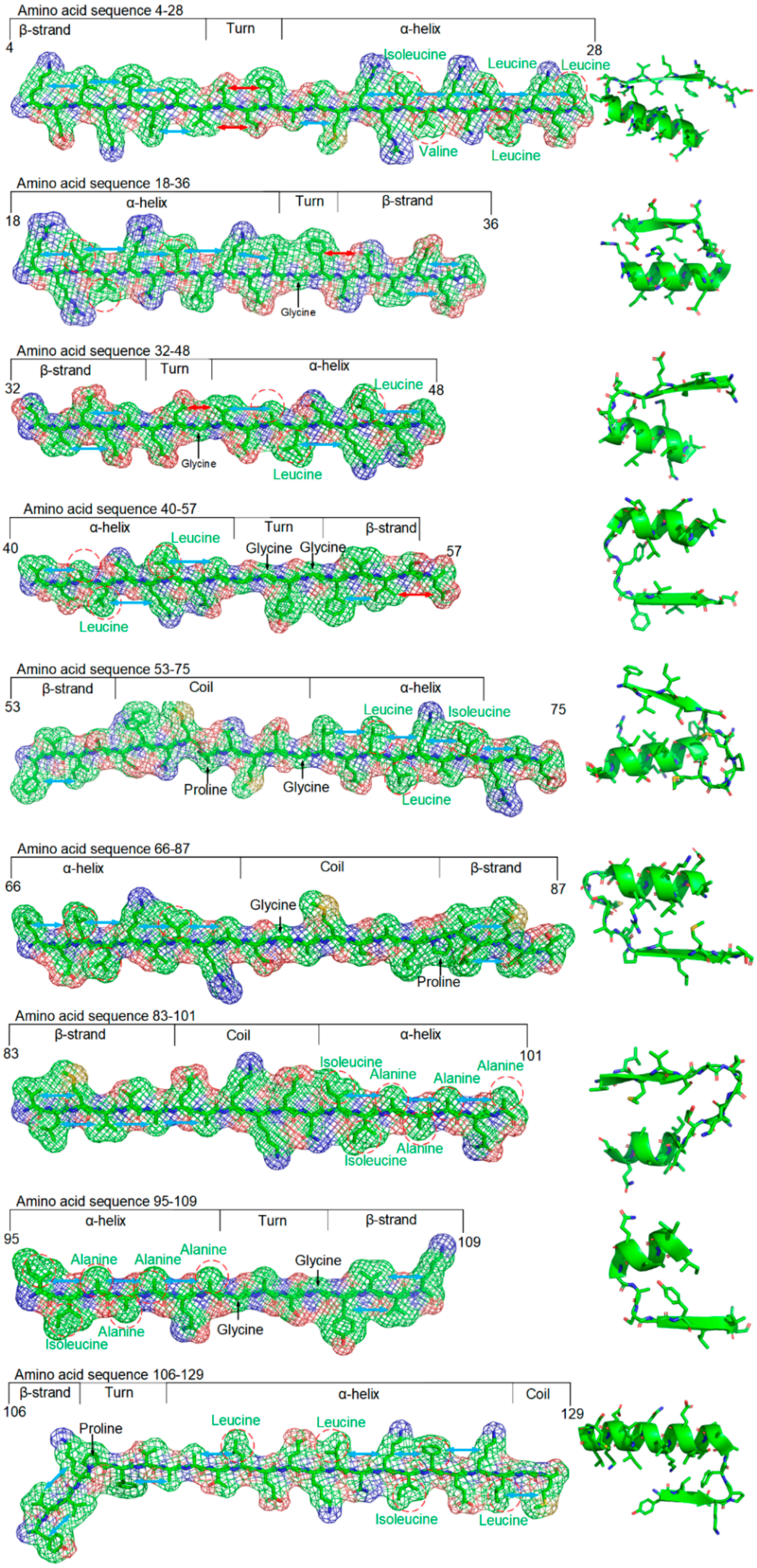

Figure 6. The folding mechanism of a protein structure (PBDID: 1CHN) based on entropy-enthalpy compensation (the hydrophobic attraction between the side-chains of adjacent residues is marked with a blue arrow, and the hydrophilic-hydrophobic repulsion is marked with a red arrow). The proline and glycine that led to the formation of the corner structure are marked. The hydrophobic amino acids in the sequence that cause the metastable collapse to form an α-helix structure are annotated by red circles.
