# Supplementary material for: Entropy-Enthalpy Compensations Fold Proteins in Precise Ways
Source: Int J Mol Sci. 2021 Sep 6;22(17):9653. doi: 10.3390/ijms22179653 (PMC8431812; doi:10.3390/ijms22179653)
Supplement: Supplementary file 1 [file ijms-22-09653-s001.zip › Supplementary6.pdf]

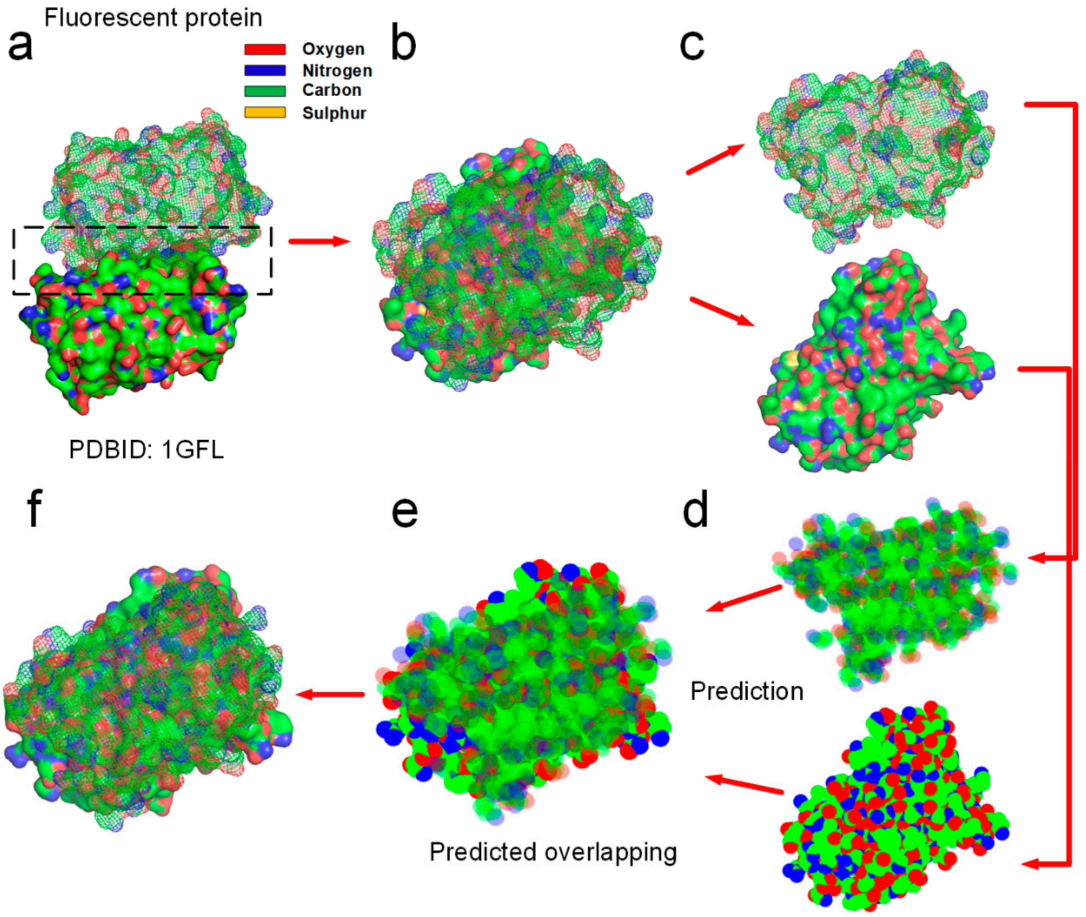

Figure 1. Prediction of the docking position between two protein subunits of the fluorescent protein dimer (PDBID:1GFL) in two dimensions by using entropy-enthalpy compensation mechanism. (a) The fluorescent protein dimer. (b) Distribution of hydrophobic (green areas) and hydrophilic (red and blue areas) surface areas on the two protein subunits at the docking sit. (c) and (d) Projective images of distribution of hydrophobic and hydrophilic surface areas at the binding site. (e) The predicted maximized the overlapping of hydrophobic surface areas of the two projective images of the two protein subunits. (f) The prediction of the docking position between the two protein subunits in two dimensions.

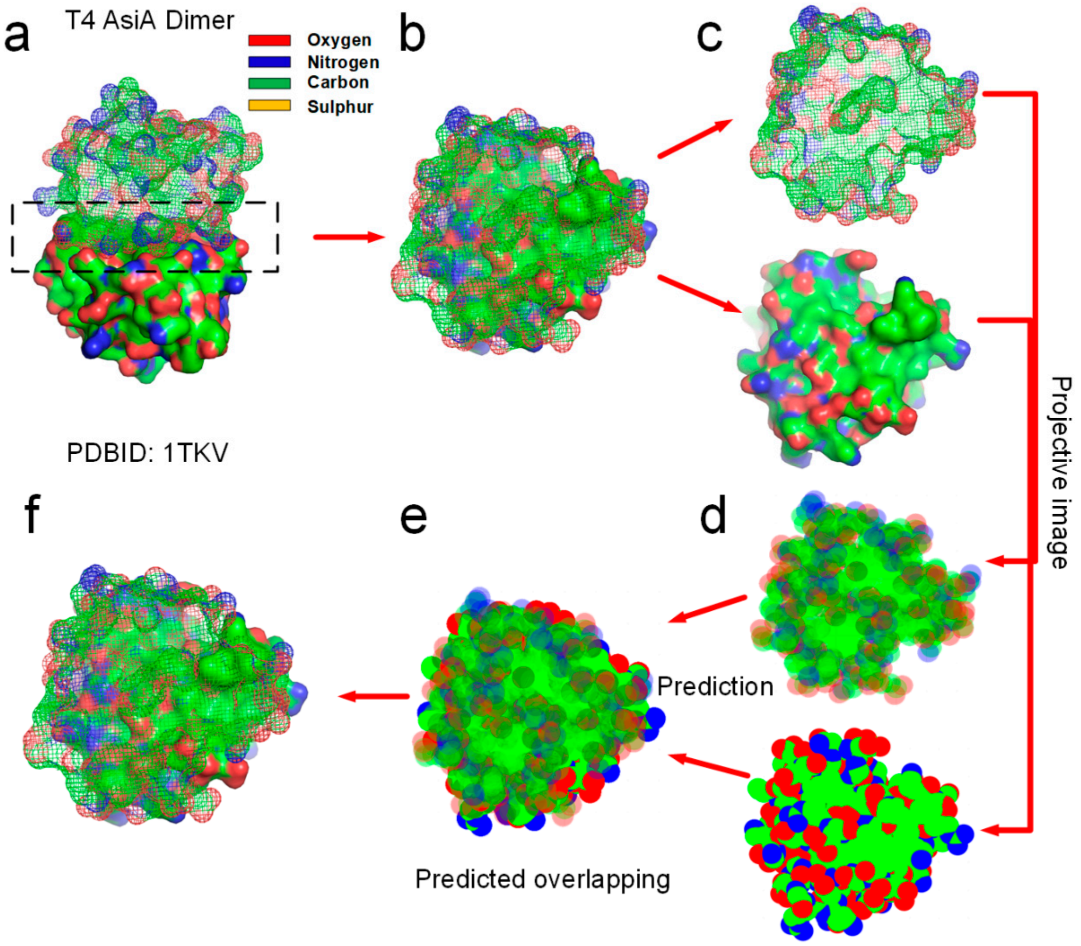

Figure 2. Prediction of the docking position between two protein subunits of the bacteriophage T4 AsiA protein dimer (PDBID: 1TKV) in two dimensions by using entropy-enthalpy compensation mechanism. (a) The bacteriophage T4 AsiA protein dimer. (b) Distribution of hydrophobic (green areas) and hydrophilic (red and blue areas) surface areas on the two protein subunits at the docking sit. (c) and (d) Projective images of distribution of hydrophobic and hydrophilic surface areas at the binding site. (e) The predicted maximized the overlapping of hydrophobic surface areas of the two projective images of the two protein subunits. (f) The prediction of the docking position between the two protein subunits in two dimensions.

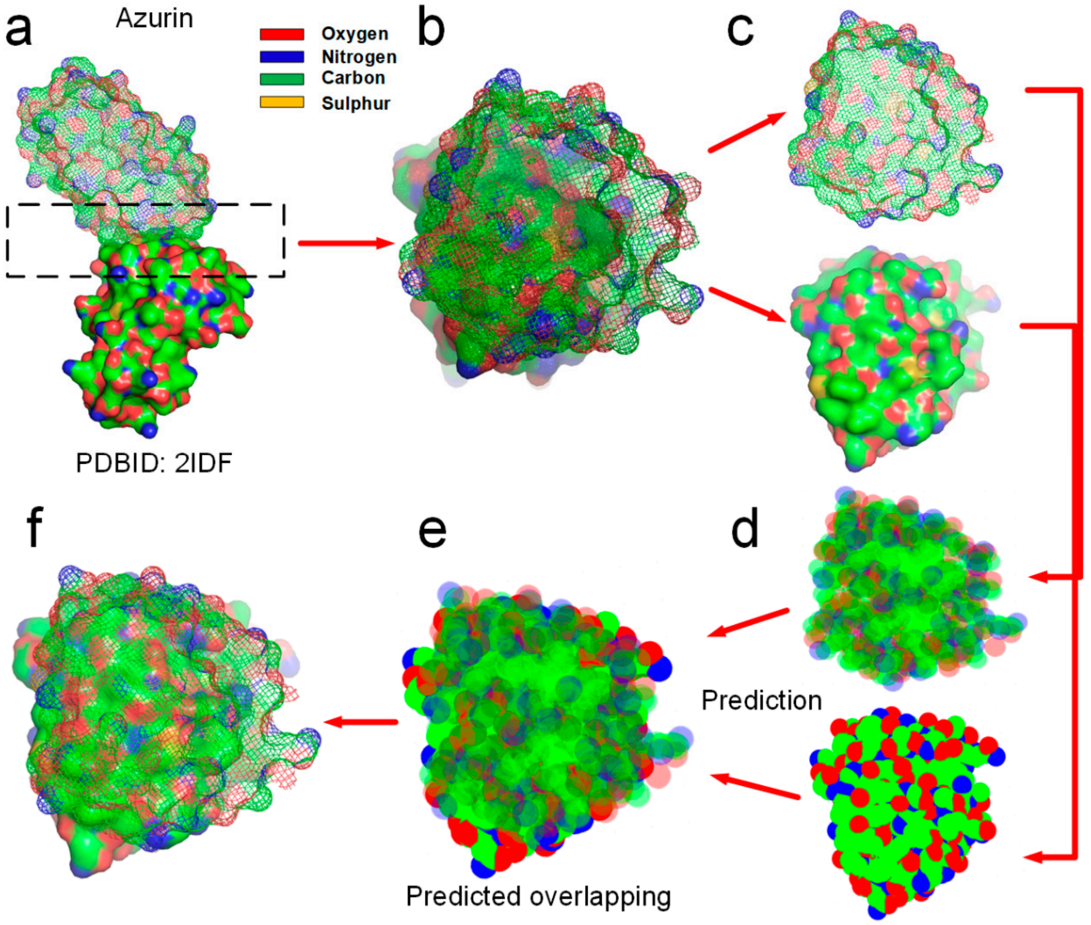

Figure 3. Prediction of the docking position between two protein subunits of the azurin protein dimer (PDBID: 2IDF) in two dimensions by using entropy-enthalpy compensation mechanism. (a) The azurin protein dimer. (b) Distribution of hydrophobic (green areas) and hydrophilic (red and blue areas) surface areas on the two protein subunits at the docking sit. (c) and (d) Projective images of distribution of hydrophobic and hydrophilic surface areas at the binding site. (e) The predicted maximized the overlapping of hydrophobic surface areas of the two projective images of the two protein subunits. (f) The prediction of the docking position between the two protein subunits in two dimensions.

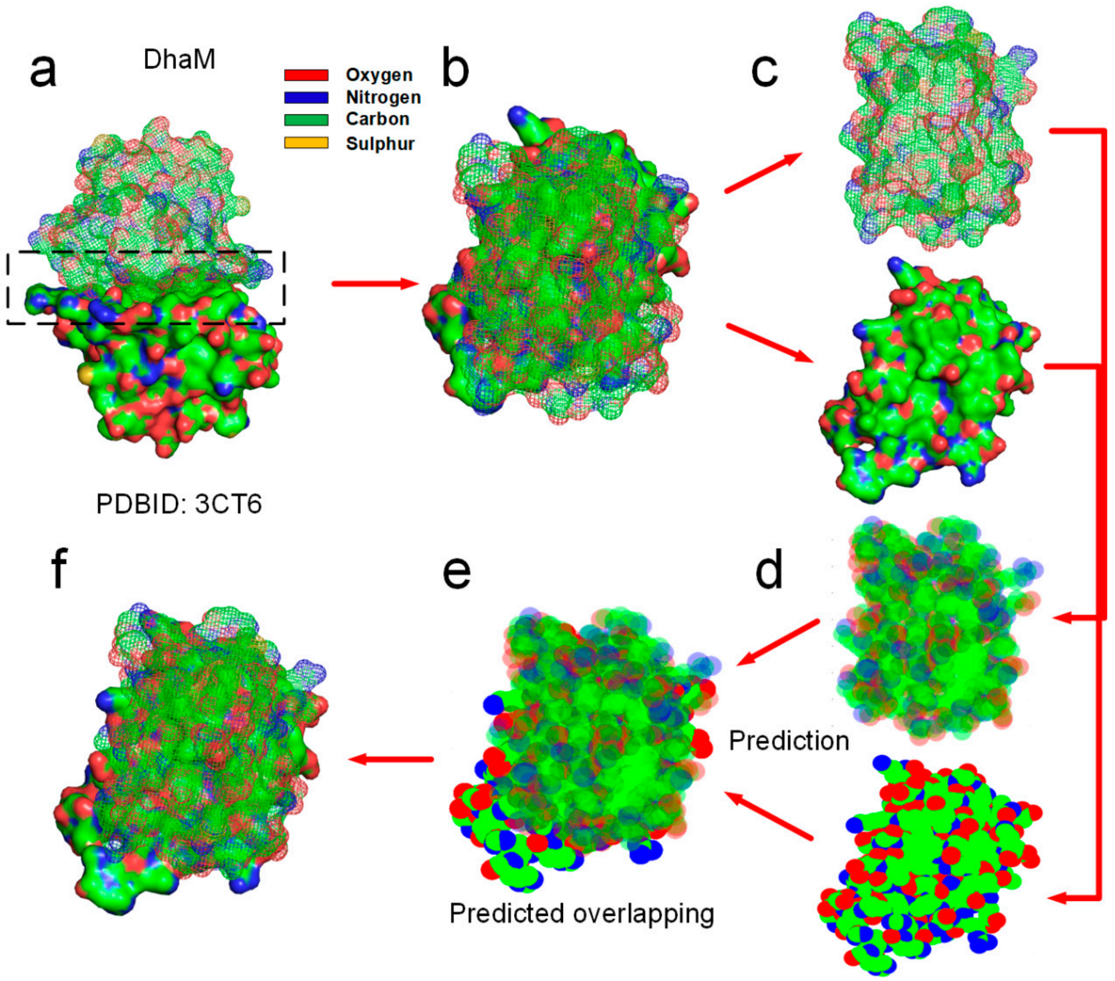

Figure 4. Prediction of the docking position between two protein subunits of the DhaM protein dimer (PDBID: 3CT6) in two dimensions by using entropy-enthalpy compensation mechanism. (a) The DhaM protein dimer. (b) Distribution of hydrophobic (green areas) and hydrophilic (red and blue areas) surface areas on the two protein subunits at the docking sit. (c) and (d) Projective images of distribution of hydrophobic and hydrophilic surface areas at the binding site. (e) The predicted maximized the overlapping of hydrophobic surface areas of the two projective images of the two protein subunits. (f) The prediction of the docking position between the two protein subunits in two dimensions.

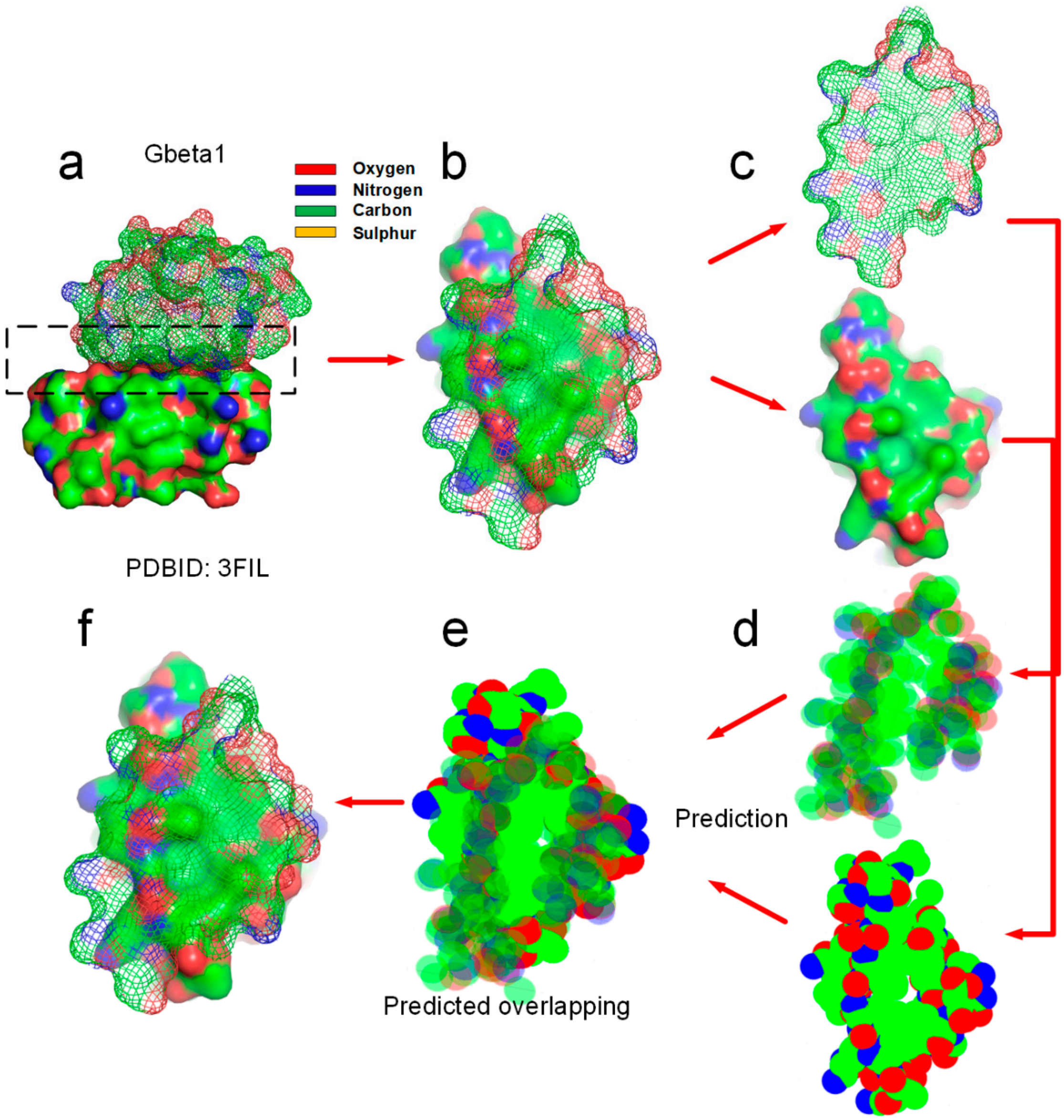

Figure 5. Prediction of the docking position between two protein subunits of the Gbeta1 protein dimer (PDBID: 3FIL) in two dimensions by using entropy-enthalpy compensation mechanism. (a) The Gbeta1 protein dimer. (b) Distribution of hydrophobic (green areas) and hydrophilic (red and blue areas) surface areas on the two protein subunits at the docking sit. (c) and (d) Projective images of distribution of hydrophobic and hydrophilic surface areas at the binding site. (e) The predicted maximized the overlapping of hydrophobic surface areas of the two projective images of the two protein subunits. (f) The prediction of the docking position between the two protein subunits in two dimensions.

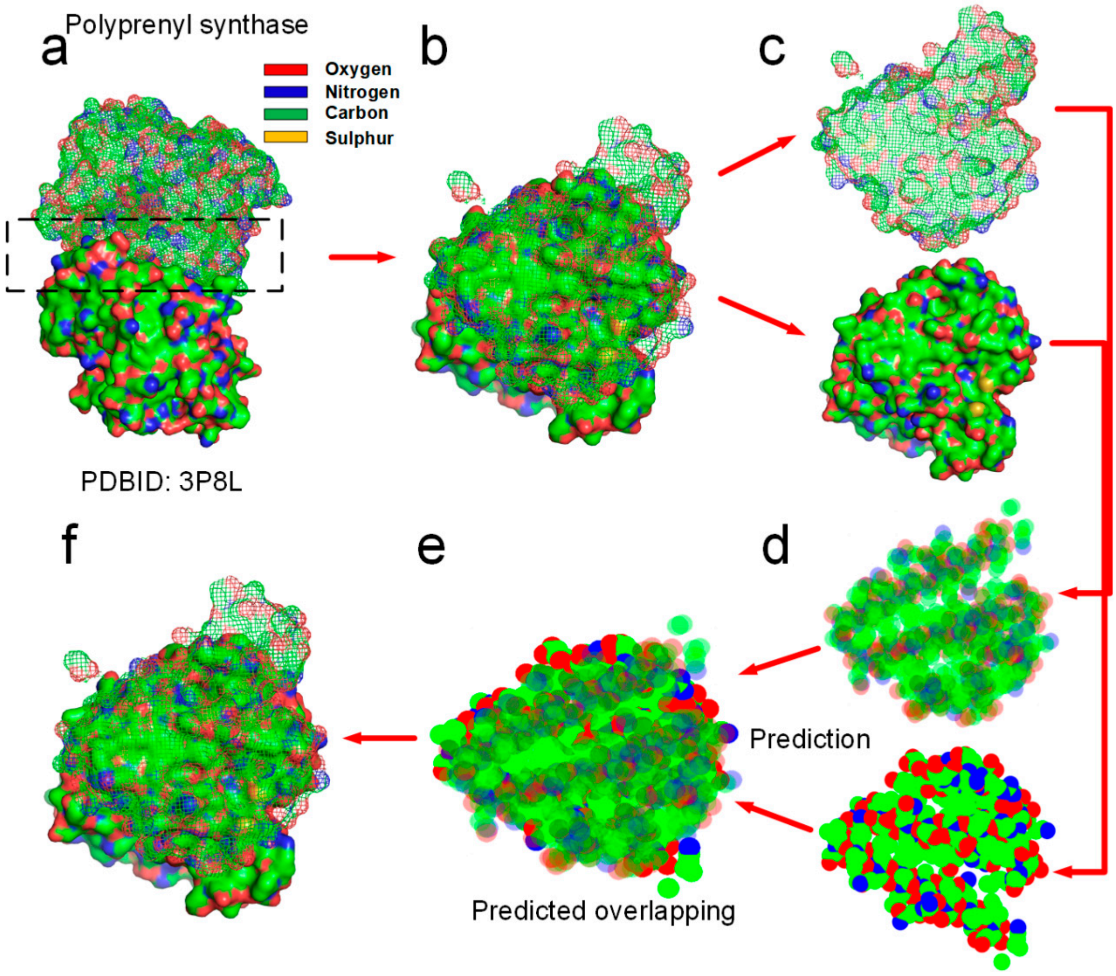

Figure 6. Prediction of the docking position between two protein subunits of the polyprenyl synthase protein dimer (PDBID: 3P8L) in two dimensions by using entropy-enthalpy compensation mechanism. (a) The polyprenyl synthase protein dimer. (b) Distribution of hydrophobic (green areas) and hydrophilic (red and blue areas) surface areas on the two protein subunits at the docking sit. (c) and (d) Projective images of distribution of hydrophobic and hydrophilic surface areas at the binding site. (e) The predicted maximized the overlapping of hydrophobic surface areas of the two projective images of the two protein subunits. (f) The prediction of the docking position between the two protein subunits in two dimensions.

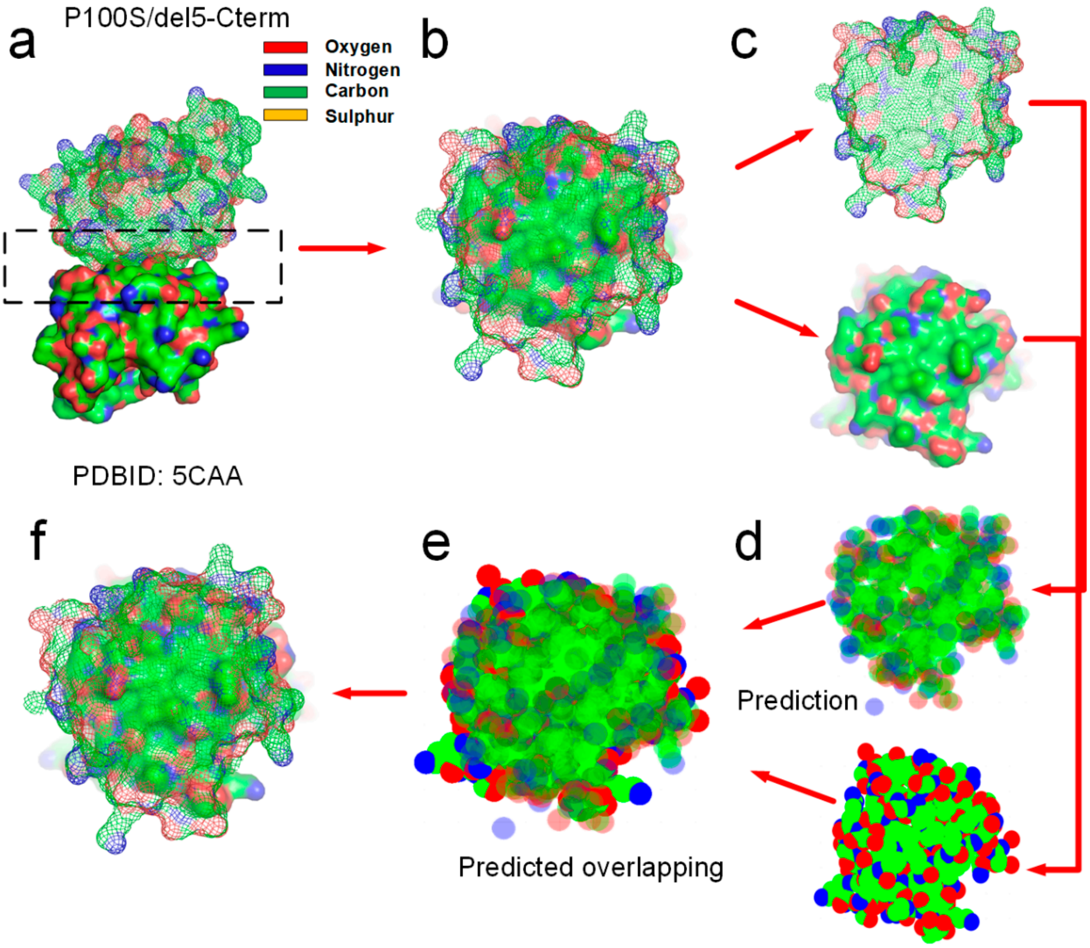

Figure 7. Prediction of the docking position between two protein subunits of the nucleoside diphosphate kinase protein dimer (PDBID: 5CAA) in two dimensions by using entropy-enthalpy compensation mechanism. (a) The nucleoside diphosphate kinase protein dimer. (b) Distribution of hydrophobic (green areas) and hydrophilic (red and blue areas) surface areas on the two protein subunits at the docking sit. (c) and (d) Projective images of distribution of hydrophobic and hydrophilic surface areas at the binding site. (e) The predicted maximized the overlapping of hydrophobic surface areas of the two projective images of the two protein subunits. (f) The prediction of the docking position between the two protein subunits in two dimensions.

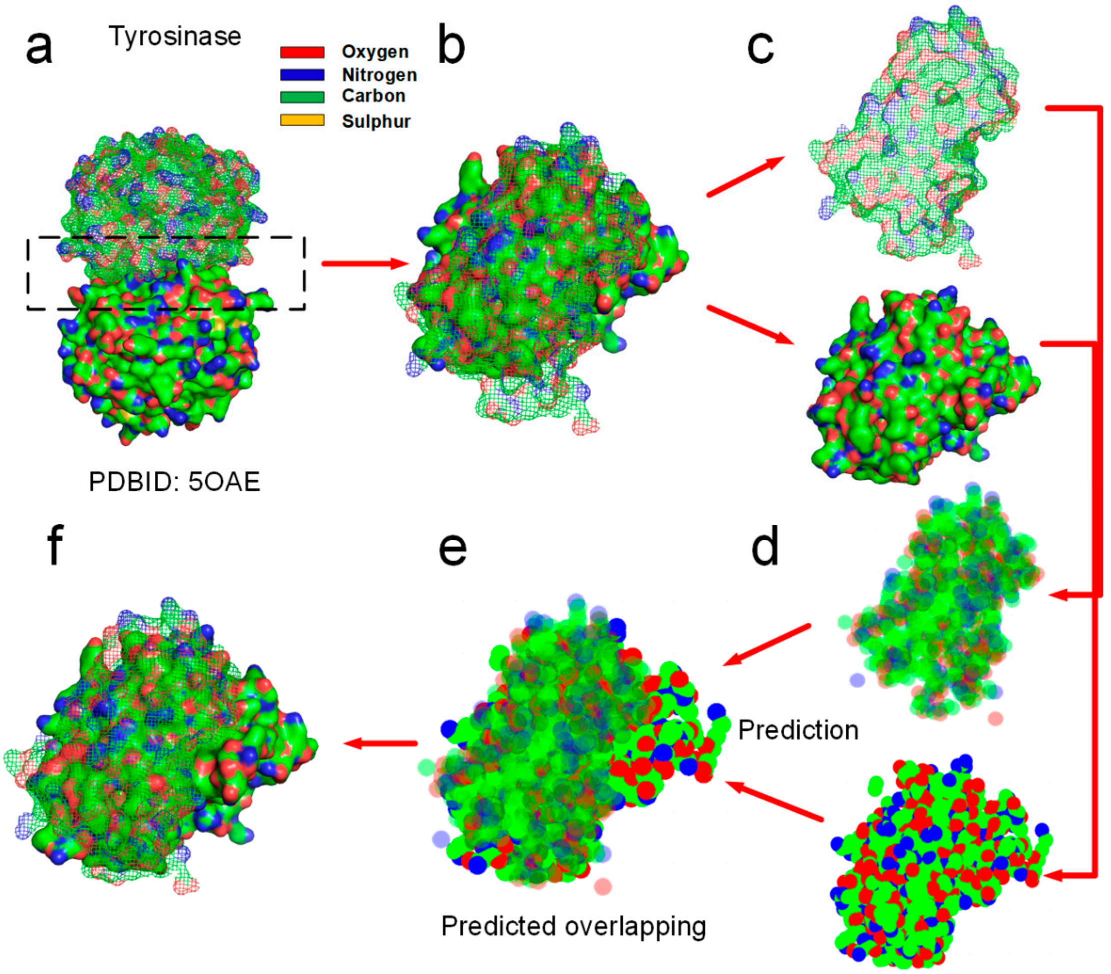

Figure 8. Prediction of the docking position between two protein subunits of the tyrosinase protein dimer (PDBID: 5OAE) in two dimensions by using entropy-enthalpy compensation mechanism. (a) The tyrosinase protein dimer. (b) Distribution of hydrophobic (green areas) and hydrophilic (red and blue areas) surface areas on the two protein subunits at the docking sit. (c) and (d) Projective images of distribution of hydrophobic and hydrophilic surface areas at the binding site. (e) The predicted maximized the overlapping of hydrophobic surface areas of the two projective images of the two protein subunits. (f) The prediction of the docking position between the two protein subunits in two dimensions.

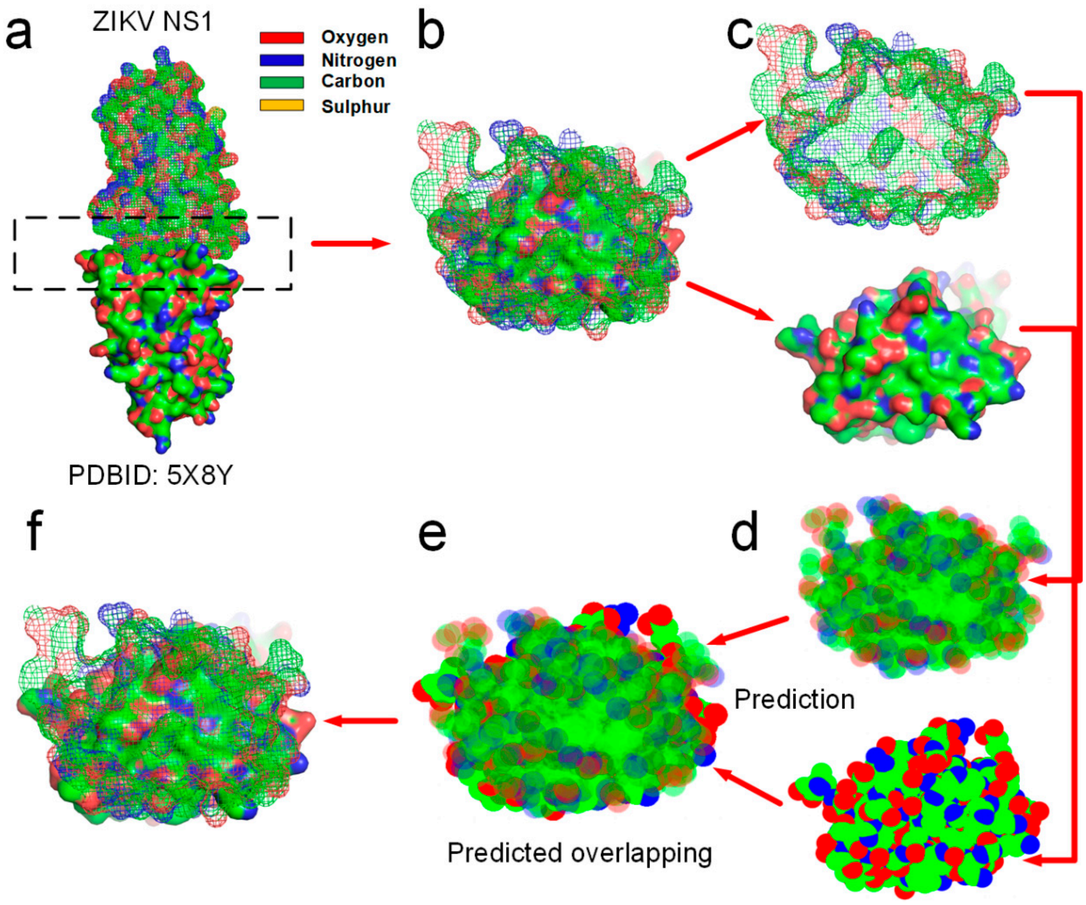

Figure 9. Prediction of the docking position between two protein subunits of the Zika virus dimer (PDBID: 5X8Y) in two dimensions by using entropy-enthalpy compensation mechanism. (a) The Zika virus protein dimer. (b) Distribution of hydrophobic (green areas) and hydrophilic (red and blue areas) surface areas on the two protein subunits at the docking sit. (c) and (d) Projective images of distribution of hydrophobic and hydrophilic surface areas at the binding site. (e) The predicted maximized the overlapping of hydrophobic surface areas of the two projective images of the two protein subunits. (f) The prediction of the docking position between the two protein subunits in two dimensions.

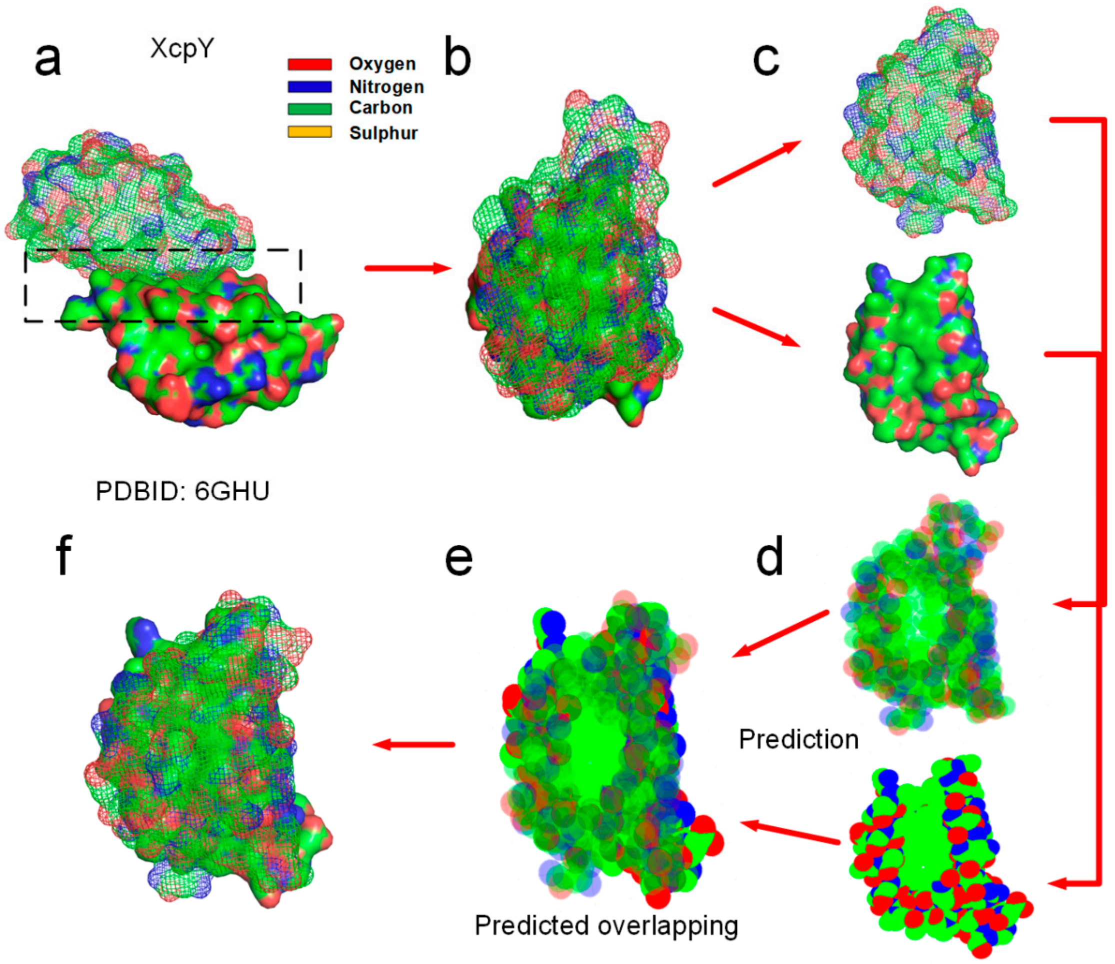

Figure 10. Prediction of the docking position between two protein subunits of the periplasmic domain dimer (PDBID: 6GHU) in two dimensions by using entropy-enthalpy compensation mechanism. (a) The periplasmic domain protein dimer. (b) Distribution of hydrophobic (green areas) and hydrophilic (red and blue areas) surface areas on the two protein subunits at the docking sit. (c) and (d) Projective images of distribution of hydrophobic and hydrophilic surface areas at the binding site. (e) The predicted maximized the overlapping of hydrophobic surface areas of the two projective images of the two protein subunits. (f) The prediction of the docking position between the two protein subunits in two dimensions.

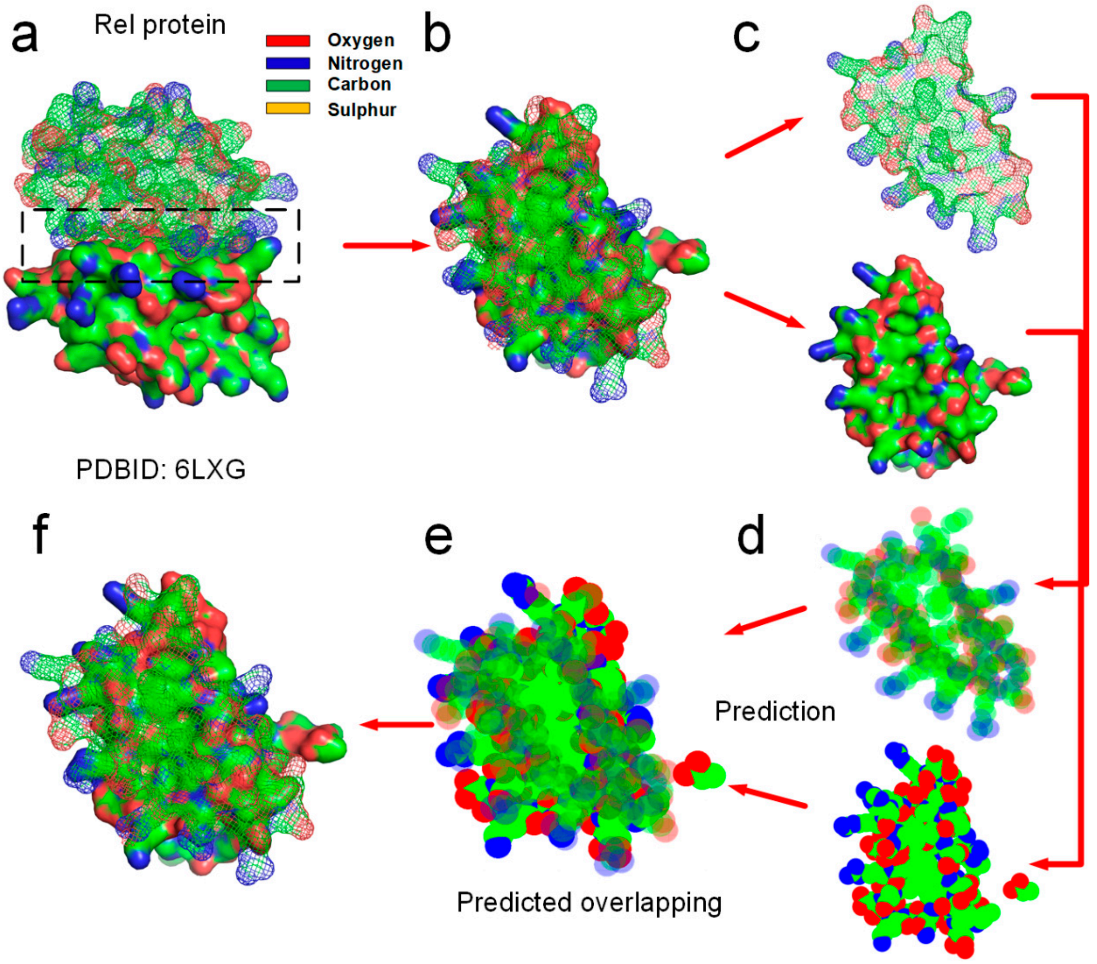

Figure 11. Prediction of the docking position between two protein subunits of the regulatory ACT domain dimer (PDBID: 6LXG) in two dimensions by using entropy-enthalpy compensation mechanism. (a) The regulatory ACT domain protein dimer. (b) Distribution of hydrophobic (green areas) and hydrophilic (red and blue areas) surface areas on the two protein subunits at the docking sit. (c) and (d) Projective images of distribution of hydrophobic and hydrophilic surface areas at the binding site. (e) The predicted maximized the overlapping of hydrophobic surface areas of the two projective images of the two protein subunits. (f) The prediction of the docking position between the two protein subunits in two dimensions.
